# Understanding the implications of under-reporting, vaccine efficiency and social behavior on the post-pandemic spread using physics informed neural networks: A case study of China

**DOI:** 10.1371/journal.pone.0290368

**Published:** 2023-11-16

**Authors:** Samiran Ghosh, Alonso Ogueda-Oliva, Aditi Ghosh, Malay Banerjee, Padmanabhan Seshaiyer

**Affiliations:** 1 Indian Institute of Technology Kanpur, Kanpur, India; 2 George Mason University, Fairfax, VA, United States of America; 3 Texas A&M University-Commerce, Commerce, TX, United States of America; Fiji National University, FIJI

## Abstract

In late 2019, the emergence of COVID-19 in Wuhan, China, led to the implementation of stringent measures forming the zero-COVID policy aimed at eliminating transmission. Zero-COVID policy basically aimed at completely eliminating the transmission of COVID-19. However, the relaxation of this policy in late 2022 reportedly resulted in a rapid surge of COVID-19 cases. The aim of this work is to investigate the factors contributing to this outbreak using a new SEIR-type epidemic model with time-dependent level of immunity. Our model incorporates a time-dependent level of immunity considering vaccine doses administered and time-post-vaccination dependent vaccine efficacy. We find that vaccine efficacy plays a significant role in determining the outbreak size and maximum number of daily infected. Additionally, our model considers under-reporting in daily cases and deaths, revealing their combined effects on the outbreak magnitude. We also introduce a novel Physics Informed Neural Networks (PINNs) approach which is extremely useful in estimating critical parameters and helps in evaluating the predictive capability of our model.

## 1 Introduction

COVID-19, caused by the novel coronavirus SARS-CoV-2, first emerged in Wuhan, Hubei province, China, in late 2019. As the virus continued to spread, China implemented stringent measures to control its transmission. Since then, China continued with the same methods, making them part of its zero-COVID policy, even after the availability of vaccines. Zero-COVID policy refers to an approach aimed at completely eliminating the transmission of COVID-19 within a specific region or country. This approach typically involves strict measures such as extensive testing, contact tracing, quarantine, travel restrictions and lockdowns to prevent the spread of the virus.

The zero-COVID policy in China has both positive and negative aspects. It aims to control the disease, protect public health, prevent healthcare system overload, and maintain economic stability. However, it also leads to economic disruptions, social and mental health impacts, potential human rights concerns, and faced challenges in feasibility and sustainability [1]. At the end of 2022, when the zero-COVID policy was relaxed gradually in China, it faced a rapid spread of the infection [2]. While we do not have access to official real-time data, there have been reports in the media suggesting a significant increase in daily COVID-19 cases in China after the relaxation of their zero-COVID policy [3–6]. According to these sources, the number of cases has allegedly surged to the magnitude of approximately ten millions per day [3, 4]. This sudden surge in epidemic progression forced researchers to think about the possible reasons behind it.

The implementation of a zero-COVID policy aims to eliminate the transmission of COVID-19, resulting in fewer infections. However, this approach may limit the development of acquired immunity in the population, leaving a significant portion susceptible to the infection. An increase in the total level of immunity within a population results in a reduction of the proportion of individuals who are susceptible to the disease. The total level of immunity in a population comes from two sources:

1. Vaccine induced immunity: Immunity obtained through vaccination of the individuals.
2. Acquired Immunity: ‘Acquired immunity’ refers to the adaptive immunity developed within a recovered individual after recovery from the infection.

Since, zero-COVID policy resulted in a fewer infections, the contribution of the acquired immunity in the total level of immunity is negligible. Consequently, the proportion of susceptible individuals is comparatively high. The vaccine induced immunity is the major contributor to reduce the proportion of susceptible population. However, the waning of immunity can increase the proportion of susceptibility over the time. This waning of immunity solely depends upon the efficacy of the vaccine. In the context of COVID-19, the level of immunity acquired through prior infection is long lasting compared to the immunity obtained through vaccination.

Vaccine efficacy refers to the effectiveness of a vaccine in preventing a disease. It is typically expressed as a percentage and represent the reduction in disease incidence among vaccinated individuals compared to a similar group of unvaccinated individuals. In the context of COVID-19, multiple vaccines have been developed in different countries by different manufacturers. Different COVID-19 vaccines employ different mechanisms to trigger an immune response. For example, mRNA based vaccines, such as those developed by Pfizer-BioNTech and Moderna, introduce genetic material into cells to produce viral proteins. In contrast, vector-based vaccines like the AstraZenca and Johnson & Johnson vaccines use weakened viruses to deliver viral proteins. These variations in vaccine mechanisms can lead to different types of vaccine efficacies [7, 8].

There were several domestic vaccine candidates in China, among which, most notably, China used Sinopharm-Beijing, Sinopharm-Wuhan, Sinovac, CanSinoBio [1, 9, 10]. The efficacies of these domestic vaccines are less as compared to other vaccines like Pfizer, Moderna, Novavax etc. (see Fig 2 in [11]). Vaccine efficacy can also be a reason behind such a large epidemic peak in the beginning of 2023 in China. Vaccine efficacy, along with its long-lasting protection, plays a crucial role in shaping the course of a new outbreak. A highly effective vaccine not only prevents infection and reduces illness, severity, but also provides durable immunity. Long-lasting vaccine efficacy ensures that individuals remain protected against the disease for an extended period, thereby contributing to the sustained control of the epidemic. By maintaining high level of immunity in the population, the vaccine helps prevent resurgence of the virus.

Under-reporting in daily cases and death data refers to the situation where the actual number of cases or deaths due to the disease is higher than what is officially reported. Under-reporting in daily cases and deaths in China has been a subject of concern and scrutiny, particularly, during disease outbreaks [12, 13]. This can have a significant impact on the progression of an epidemic by influencing human behavior. When the true magnitude of an epidemic is not accurately reflected in the official statistics, it can create a false perception of the severity of the situation. This can lead to a sense of complacency or decreased vigilance among the population, as individuals may underestimate the risk of infection and adopt less stringent preventive measures. Consequently, under-reporting can result in increased transmission of the virus as people may be less likely to adhere to essential precautions. This can further fuel the spread of the epidemic. Accurate and transparent reporting of daily cases and deaths is crucial to ensure that individuals maintain a realistic understanding of the situation, leading to appropriate behavior that helps curb the transmission of the virus.

In our study, we aim to investigate the factors contributing to a large outbreak, such as the one observed in China, in the beginning of 2023, and to achieve this, we develop a modified SEIR epidemic model [14–16]. One of the important component of our SEIR-type model is that the model incorporates a time-dependent level of immunity, considering both the number of vaccine doses administered and the efficacy of the vaccine. Notably, we consider the vaccine efficacy as a function of the time-post-vaccination because the efficacy is not same all the time after vaccination.

Our computational results demonstrate that the post-vaccination dependent vaccine efficacy plays a significant role in determining the size of the epidemic outbreak. Furthermore, we enhance the precision of our model by integrating *imitation dynamics* into our framework. These dynamics capture individuals’ behavior in switching strategies for reporting infections. We also consider that the decision to switch reporting strategies may depend on the perception of disease severity, which is influenced by available death data. Our modelling results reveal that although under-reporting of deaths may not directly impact disease incidence, it can create a false perception of reduced disease severity, leading to implicit under-reporting of daily cases. The combined effects of under-reporting in daily cases and deaths can influence the magnitude of the outbreak.

Along with new ideas on modeling, we also introduce neural network based approaches to quantify parameters in the model learning from the data. Specifically, we use Physics Informed Neural Networks (PINNs) approaches that have been applied to a variety of differential equations [17–19]. Building on this, we also delve into the development of an algorithm based on PINNs in this work applied to the system of differential equations proposed. This algorithm serves the purpose of estimating critical parameters that play a crucial role in understanding the dynamics of the epidemic.

Approaches such as PINNs helps with the realization of the need for artificial intelligence and machine learning (AI/ML) modeling platforms that facilitate discovery of novel biological phenomena, rules, and theories. This work aligns with calls from funding agencies such as the US National Science Foundation that promote the need for mathematical, computational, and biological scientists to work together to develop MODels for Uncovering Rules and Unexpected Phenomena in Biological Systems (MODULUS (https://www.nsf.gov/pubs/2021/nsf21069/nsf21069.jsp)) as well as incorporating human behavior in epidemiological models (IHBEM (https://www.nsf.gov/pubs/2023/nsf23546/nsf23546.htm)).

**The goals of our study can be summarized as follows:**

- Develop a new SEIR-type epidemic model with time-dependent level of immunity, considering vaccine doses administrated and the time-post-vaccination dependent vaccine efficacy,
- Analyze the significant role of time-post-vaccination dependent vaccine efficacy in determining the size of the outbreak,
- Explore the explicit and implicit effects of the under-reporting in daily cases and deaths on the magnitude of the outbreak and,
- Apply a predictive algorithm to estimate important parameters for understanding the epidemic progression.

The content of the paper is as follows. We discuss the data sources, model formulation and calculate the basic epidemic quantities of the model in section (2). In section (3), we introduce under-reporting in infected and death in the model formulation. Parameter estimation using Physics Informed Neural Networks (PINNs) is discussed in section (4). Computational results are discussed in section (5).

## 2 Materials and methods

### 2.1 Data sources

**Fig 1:**
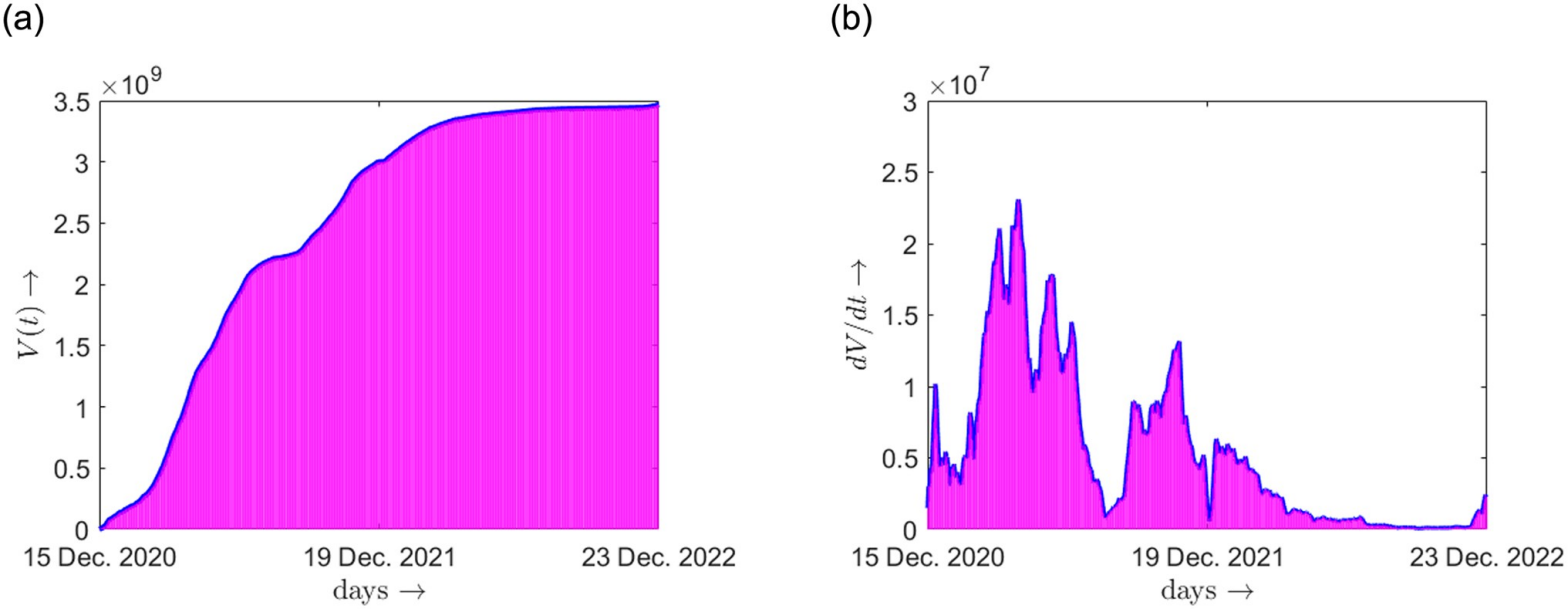
Real data of vaccination in China [10]. (a) Cumulative vaccination data *V*(*t*), (b) Daily vaccination data which approximately gives *dV*/*dt*.

**Fig 2:**
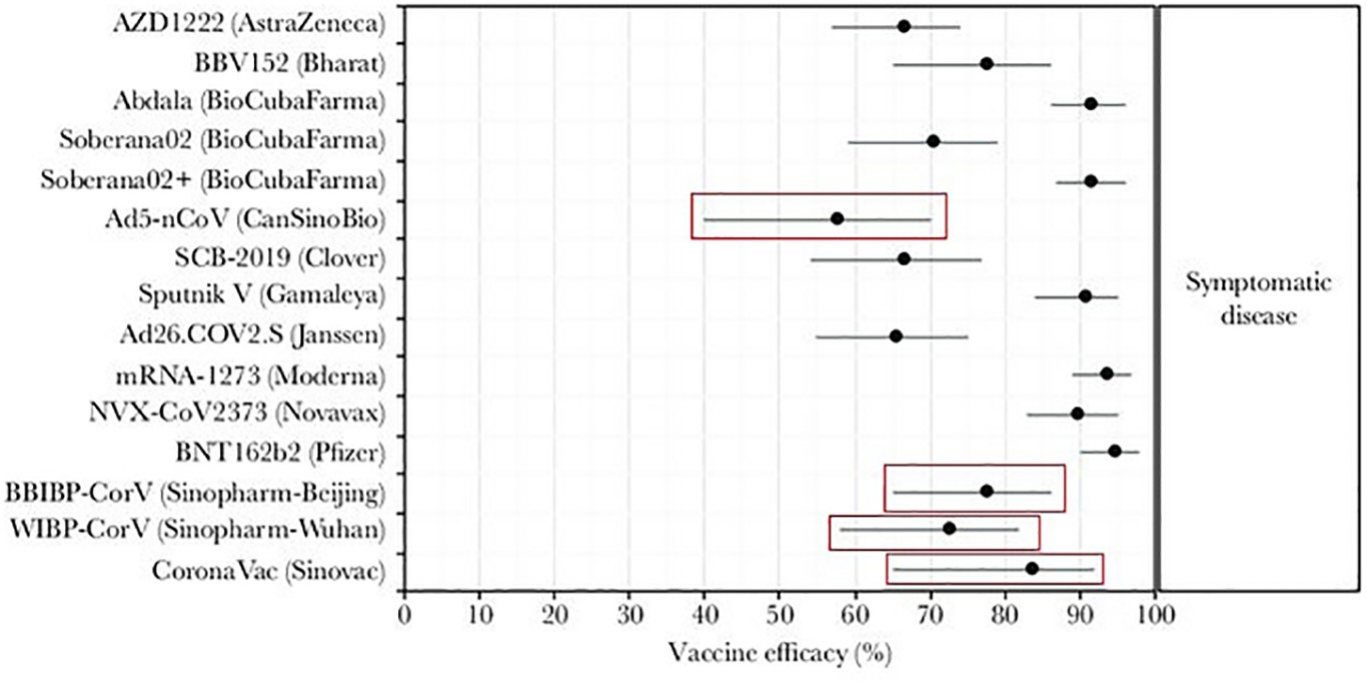
Comparison among the effectiveness of different vaccines of COVID-19. In China, mainly Sinopharm-Bejing, Sinopharm-Wuhan, Sinovac, CanSinoBio vaccines were administrated (the red squares) [1, 9, 10]. This figure is taken from [11].

We first describe the data sources utilized in our current study. To examine the vaccination administration in China, we collected the time-series data of vaccination in China, from [10]. The trend in vaccination coverage can be visualized from the Fig 1. Furthermore, to support our modelling results, we gathered data from a secondary source [11], which presents a comparative analysis of vaccine efficacies. The visual representation of these comparisons, displayed in Fig 2, provides valuable insights into the relative effectiveness of different vaccines. Moreover, we used some reliable parameter values associated to our modelling, available in some sources, as referenced in Table 1.

**Table 1:** Parameter values.

Parameters	Description	Value	Units	Source
*N*	Total population	1.453 × 10^9^	no. of people	[20]
*β*	Transmission rate	11	day^−1^	—
*σ*	Reciprocal of incubation period	1/4.35	day^−1^	[21]
*r*	Rate at which exposed individuals become symptomatic	0.227	unit-less	[22]
*α*	Relative transmission rate of asymptomatic infection	0.43	unit-less	[23]
*η*_1_	Rate of quarantine	0.001	day^−1^	—
*η*_2_	Rate of hospitalization	0.0689	day^−1^	[24]
*ξ*	Rate of hospitalization of quarantined	0.03	day^−1^	—
*δ*_*I*_	Recovery rate of infected	0.9975	day^−1^	[25]
*μ*_*I*_	Death rate of infected	0.0025	day^−1^	[25]
*δ*_*A*_	Recovery rate of asymptomatic	0.9975	day^−1^	—
*δ*_*H*_	Recovery rate of hospitalized	0.9975	day^−1^	—
*μ*_*H*_	Death rate of hospitalized	0.0015	day^−1^	—
*δ*_*Q*_	Recovery rate of quarantined	0.9975	day^−1^	—
*V*(*t*)	Daily total vaccination data of China	Fig 1	no. of people	[10]

Note that, some of the parameters in Table 1 can be obtained from reliable sources, but, to the best of our knowledge, the rest of the parameters in Table 1 are not available in the existing literature, in the context of COVID-19 in China after the relaxation of the zero-COVID policy. On the other hand, according to some sources [3, 4], after the relaxation of the zero-COVID policy, the number of daily cases in China was in the magnitude of few millions per day. Since, detailed time series data for COVID-19 in China is not available in the literature, with the minimal information available at the sources [3, 4], we initially assumed some of the parameter values which are not available in reliable sources, as indicated in Table 1. Assumption of such parameter values is motivated by the daily number of cases obtained from our model outcome reaches the magnitude of few millions per day (e.g., green curve in Figs 17(c) and 18). However, later in this work, we employed a PINNs algorithm that helped to estimate some of those parameters involved in our model, using the initial assumptions of the parameter values.

### 2.2 Model description

Let us suppose vaccination in China was started at time *t* = *t*_0_ = 0 and the zero-COVID policy was relaxed from *t* = *t*_1_. Since there is negligible number of infection due to zero-COVID policy during the time interval [*t*_0_, *t*_1_], we neglect the acquired immunity due to infection, whereas, during the same time interval, the level of immunity in the population increased due to vaccination. Our main concern is to study the sudden large outbreak in a relatively shorter period of time starting from *t*_1_ (i.e., the time when zero-COVID policy was relaxed), and we assume that those who get recovered after time *t*_1_ do not become susceptible during our time interval of study.

Here we consider following compartments: susceptible (*S*), exposed (*E*), asymptomatic (*A*), infected (*I*), quarantined (*Q*), hospitalized (*H*), recovered (*R*) and deceased (*D*) with total population *N* is supposed to be a fixed constant.

Time dependent growth rates of each compartments, except *S* and *D* are given by

$$\begin{array}{ccc} \frac{dE}{dt} & = & \frac{\beta S}{N}\left(I+\alpha A\right)-\sigma E \end{array}$$ (1a)

$$\begin{array}{ccc} \frac{dI}{dt} & = & r\sigma E-(\eta_{1}+\eta_{2}+\delta_{I}+\mu_{I})I \end{array}$$ (1b)

$$\begin{array}{ccc} \frac{dA}{dt} & = & \left(1-r\right)\sigma E-\delta_{A}A \end{array}$$ (1c)

$$\begin{array}{ccc} \frac{dQ}{dt} & = & \eta_{1}I-\left(\xi +\delta_{Q}\right)Q \end{array}$$ (1d)

$$\begin{array}{ccc} \frac{dH}{dt} & = & \eta_{2}I+\xi Q-\left(\delta_{H}+\mu_{H}\right)H \end{array}$$ (1e)

$$\begin{array}{ccc} \frac{dR}{dt} & = & \delta_{I}I+\delta_{A}A+\delta_{H}H+\delta_{Q}Q. \end{array}$$ (1f)

Let *m*(*t*) denote the level of immunity due to vaccination given by

$$\begin{array}{ccc} m(t) & = & \frac{1}{N}\int_{0}^{t}\varphi \left(t-\eta \right)V^{\prime }\left(\eta \right)d\eta , \end{array}$$ (2)

where *V*′(*η*) is nonnegative function which represents number of daily vaccinations and *ϕ*(*η*) is the efficacy of vaccine at the time-post-vaccination *η*. **Note.** Here, the level of immunity *m*(*t*) in Eq (2) is defined only in terms of the vaccine induced immunity. This definition of *m*(*t*) does not consider the acquired immunity developed within an individual after recovering from the infection. This is because of the fact that there was negligible number of infection due to zero-COVID policy.

Then the equation for susceptible can be written as:

$$\begin{array}{ccc} S(t) & = & N-(E(t)+I(t)+A(t)+Q(t)+H(t)+R(t)+D(t)+Nm(t)). \end{array}$$ (3)

**Fig 3:**
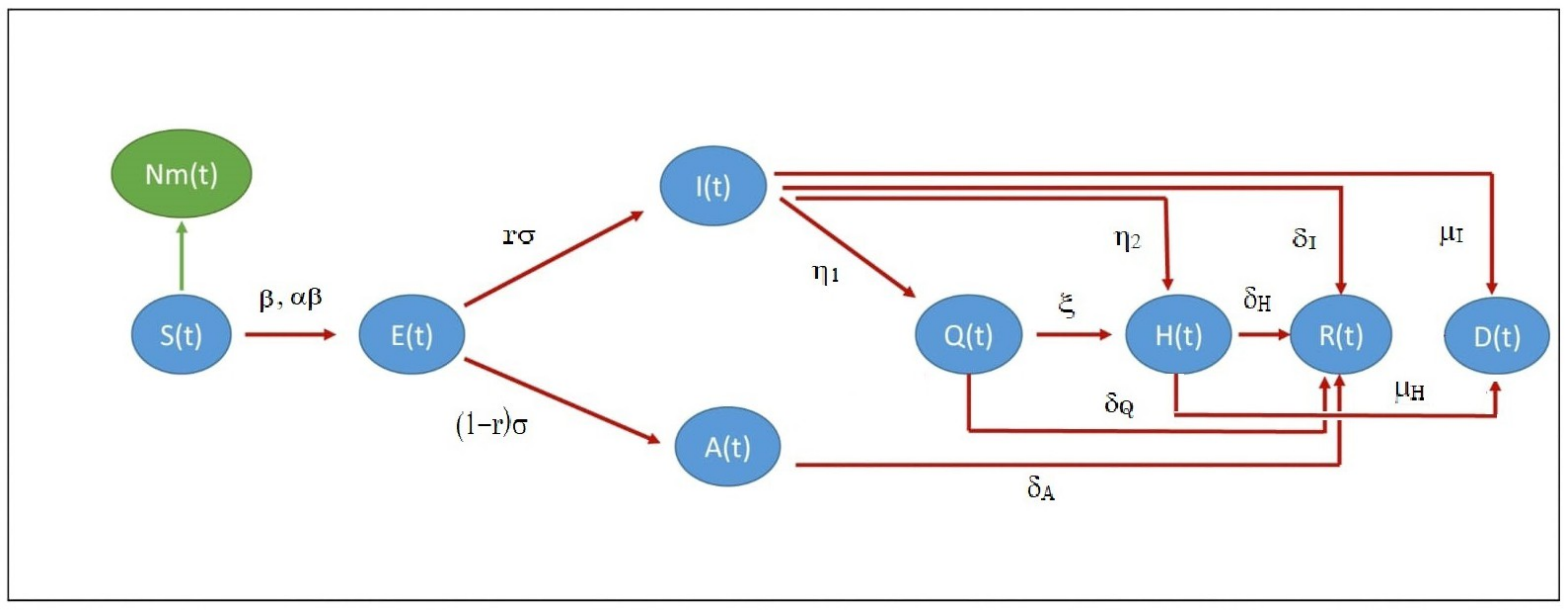
Compartmental flow diagram of the model (1).

A compartmental flow diagram of the model (1)-(3) is shown in Fig 3. Though the Eq (3) determines *S*(*t*) in terms of all other compartments, this relation does not clearly explains how the susceptible becomes exposed due to the interaction with the compartments *I* and *A*. Hence, we derive the governing differential equation for the *S* compartment which is determined by the interaction with the compartments *I*, *A* and the level of immunity *m*(*t*).

Differentiating both sides of the Eq (2) and using the Leibnitz rule for differentiation under the integral sign, we get

$$\begin{array}{ccc} \frac{dm(t)}{dt} & = & \frac{1}{N}\frac{d}{dt}\int_{0}^{t}\phi \left(t-\eta \right)V^{\prime }\left(\eta \right)d\eta \\ & = & \frac{1}{N}[\int_{0}^{t}\phi^{\prime }\left(t-\eta \right)V^{\prime }\left(\eta \right)d\eta +\phi \left(0\right)V^{\prime }\left(t\right)]. \end{array}$$

Assuming *ϕ*(0) = 0 we get

$$\begin{array}{ccc} \frac{dm(t)}{dt} & = & \frac{1}{N}\int_{0}^{t}\phi^{\prime }\left(t-\eta \right)V^{\prime }\left(\eta \right)d\eta . \end{array}$$ (4)

Now, differentiating (3) with respect to *t* and using the equations in system (1), we get,

$$\begin{array}{ccc} \frac{dS(t)}{dt} & = & -\frac{\beta S}{N}\left(I+\alpha A\right)-N\frac{dm(t)}{dt}. \end{array}$$ (5)

Substituting the expression of *dm*/*dt* from (4) in (5), we get,

$$\begin{array}{ccc} \frac{dS(t)}{dt} & = & -\frac{\beta S}{N}\left(I+\alpha A\right)-\int_{0}^{t}\phi^{\prime }\left(t-\eta \right)V^{\prime }\left(\eta \right)d\eta . \end{array}$$ (6)

The Eq (6) is the governing equation for the rate of change of the *S* compartment which clearly explains how the rate of change of the *S* compartment depends on (i) the interaction of the individuals in *S* compartment with the individuals in the compartments *I*, *A*, (ii) the vaccine efficacy function *ϕ*(*t*) and (iii) the number of vaccinated individuals *V*(*t*).

The growth rate of *D*(*t*) is given by

$$\begin{array}{ccc} \frac{dD}{dt} & = & \mu_{I}I+\mu_{H}H. \end{array}$$ (7)

Hence the full system can be written as follows:

$$\begin{array}{ccc} \frac{dS}{dt} & = & -\frac{\beta S}{N}\left(I+\alpha A\right)-\int_{0}^{t}\phi^{\prime }\left(t-\eta \right)V^{\prime }\left(\eta \right)d\eta \end{array}$$ (8a)

$$\begin{array}{ccc} \frac{dE}{dt} & = & \frac{\beta S}{N}\left(I+\alpha A\right)-\sigma E \end{array}$$ (8b)

$$\begin{array}{ccc} \frac{dI}{dt} & = & r\sigma E-(\eta_{1}+\eta_{2}+\delta_{I}+\mu_{I})I \end{array}$$ (8c)

$$\begin{array}{ccc} \frac{dA}{dt} & = & \left(1-r\right)\sigma E-\delta_{A}A \end{array}$$ (8d)

$$\begin{array}{ccc} \frac{dQ}{dt} & = & \eta_{1}I-\left(\xi +\delta_{Q}\right)Q \end{array}$$ (8e)

$$\begin{array}{ccc} \frac{dH}{dt} & = & \eta_{2}I+\xi Q-\left(\delta_{H}+\mu_{H}\right)H \end{array}$$ (8f)

$$\begin{array}{ccc} \frac{dR}{dt} & = & \delta_{I}I+\delta_{A}A+\delta_{H}H+\delta_{Q}Q \end{array}$$ (8g)

$$\begin{array}{ccc} \frac{dD}{dt} & = & \mu_{I}I+\mu_{H}H. \end{array}$$ (8h)

### 2.3 Positivity and boundedness of solution

We assume that the vaccine efficacy function *ϕ*(*t*) is zero at *t* = 0 and monotonically increases up to $t=\tilde{t}$, and after that it is monotonically decreasing. Under this assumption we have,

$$\phi^{\prime }\left(t\right)\geq 0\;\text{for}\;0<t\leq \tilde{t},\;\text{and}\;\phi^{\prime }\left(t\right)\leq 0\;\text{for}\;t>\tilde{t}.$$

Also, we assume that when the number of susceptible is close to 0 then we can stop new vaccination. Without any loss of generality we suppose that *t* = t_*_ is the first time when *S*(t_*_) = 0. Then from the epidemiological point of view, we assume

$$\begin{array}{c} V^{\prime }\left(t\right)=0,\;\;\text{for}\;\;t\in \left[\text{max}\left\{0,t_{*}-\tilde{t}\right\},\;t_{*}\right]. \end{array}$$ (9)

Now, from (8a), we can write

$$\begin{array}{ccc} \frac{dS}{dt} & = & -\frac{\beta S}{N}\left(I+\alpha A\right)-\int_{0}^{t}\phi^{\prime }\left(t-\eta \right)V^{\prime }\left(\eta \right)d\eta \\ & = & -\frac{\beta S}{N}\left(I+\alpha A\right)-\int_{0}^{t}\phi^{\prime }\left(\eta \right)V^{\prime }\left(t-\eta \right)d\eta . \end{array}$$

Evaluating $\frac{dS}{dt}$ at *t* = t_*_, we find,

$$\begin{array}{ccc} \frac{dS}{dt}{|}_{t=t_{*}} & = & -\frac{\beta S(t_{*})}{N}\left(I\left(t_{*}\right)+\alpha A\left(t_{*}\right)\right)-\int_{0}^{t_{*}}\phi^{\prime }\left(\eta \right)V^{\prime }\left(t_{*}-\eta \right)d\eta \\ & = & -\int_{0}^{t_{*}}\phi^{\prime }\left(\eta \right)V^{\prime }\left(t_{*}-\eta \right)d\eta . \end{array}$$

Now, if $t_{*}<\tilde{t}$ then using (9) we get *V*′(*t*) = 0 when *t* ∈ [0, t_*_]. Then we get

$$\begin{array}{ccc} \frac{dS}{dt}{|}_{t=t_{*}} & = & -\int_{0}^{t_{*}}\phi^{\prime }\left(\eta \right)V^{\prime }\left(t_{*}-\eta \right)d\eta =0. \end{array}$$ (3)

If $t_{*}\geq \tilde{t}$ then using (9) we get *V*′(*t*) = 0 when $t\in [t_{*}-\tilde{t},t_{*}]$. Then we get

$$\begin{array}{ccc} \frac{dS}{dt}{|}_{t=t_{*}} & = & -\int_{0}^{\tilde{t}}\phi^{\prime }\left(\eta \right)V^{\prime }\left(t_{*}-\eta \right)d\eta -\int_{\tilde{t}}^{t_{*}}\phi^{\prime }\left(\eta \right)V^{\prime }\left(t_{*}-\eta \right)d\eta \\ & = & -\int_{\tilde{t}}^{t_{*}}\phi^{\prime }\left(\eta \right)V^{\prime }\left(t_{*}-\eta \right)d\eta \geq 0, \end{array}$$

since, *ϕ*′(*η*) ≤ 0 for $\tilde{t}\leq \eta \leq t_{*}$, and *V*′(*η*) ≥ 0 for all *η* ≥ 0. Hence in both the cases we find, $\frac{dS}{dt}{|}_{t=t_{*}}\geq 0$. This proves that if *S*(0) > 0, then *S*(*t*) ≥ 0 for all *t* > 0. Now to prove the positivity of the other compartments we follow the same approach as in [26]. Let

$$R_{+}^{7}\;=\;\{\left(E,I,A,Q,H,R,D\right)\;\in \;R^{7}\;:\;\;E,\;I,\;A,\;Q,\;H,\;R,\;D\;\geq \;0\}$$

denote the non-negative cone of $R^{7}$ and denote

$$X\left(t\right)=(E(t),I(t),A(t),Q(t),H(t),R(t),D(t))\in R_{+}^{7}.$$

Then we have the following inequalities:

$${\frac{dE}{dt}|}_{E=0,X\left(t\right)\in R_{+}^{7}}\;=\;\frac{\beta S}{N}\left(I+\alpha A\right)\;\geq \;0,\;\;{\frac{dI}{dt}|}_{I=0,X\left(t\right)\in R_{+}^{7}}\;=\;r\sigma E\;\geq \;0,\;\;$$

$${\frac{dA}{dt}|}_{A=0,X\left(t\right)\in R_{+}^{7}}\;=\;\left(1-r\right)\sigma E\;\geq \;0,\;\;{\frac{dQ}{dt}|}_{Q=0,X\left(t\right)\in R_{+}^{7}}\;=\;\eta_{1}I\;\geq \;0,\;\;$$

$${\frac{dH}{dt}|}_{H=0,X\left(t\right)\in R_{+}^{7}}\;=\;\eta_{2}I+\xi Q\;\geq \;0,\;\;{\frac{dR}{dt}|}_{R=0,X\left(t\right)\in R_{+}^{7}}\;=\;\delta_{I}I+\delta_{A}A+\delta_{H}H+\delta_{Q}Q\;\geq \;0,$$

$${\frac{dD}{dt}|}_{D=0,X\left(t\right)\in R_{+}^{7}}\;=\;\mu_{I}I+\mu_{H}H\;\geq \;0.$$

This ensures that if we start with a non-negative initial condition then the system is invariant in $R_{+}^{7}.$ Now using the Eq (3) we can write,

0≤S(t)+E(t)+I(t)+A(t)+Q(t)+H(t)+R(t)+D(t)≤N,

for all time *t* ≥ 0, which proves the boundedness of solution of the system (8).

### 2.4 Controlled reproduction number

In this article we are considering the situation not from the onset of epidemic progression rather we are starting from an intermediate time point. Hence we will be interested on effective reproduction number rather than so called basic reproduction number. Further, at the targeted initial time the vaccination drive is already started and hence the obtained threshold will be somewhat controlled reproduction number.

Suppose vaccination in China was started at time *t* = *t*_0_ = 0 and the zero-COVID policy was relaxed from *t* = *t*_1_, and since, in China there was negligible infection as compared to the total population during [*t*_0_, *t*_1_], we neglect infections, recovery and death in the interval [*t*_0_, *t*_1_], and we suppose the infection enters the population at time *t* = *t*_1_. Hence our interest is to determine the reproduction number at time *t* = *t*_1_. Consider the equilibrium point (*S*(*t*_1_), 0, 0, 0, 0, 0, 0, 0). Now,

$$\begin{array}{c} S\left(t_{1}\right)\;=\;N(1-m(t_{1}))\;=\;N-\int_{0}^{t_{1}}\phi \left(t_{1}-\eta \right)V^{\prime }\left(\eta \right)d\eta . \end{array}$$

We use the next generation matrix approach [27], to calculate the controlled reproduction number the details of which is in Sec.7.

The controlled reproduction number $R_{c}$ at time *t* = *t*_1_ is given by:

$$\begin{array}{ccc} R_{c} & = & \frac{\beta S(t_{1})r}{N(\eta_{1}+\eta_{2}+\delta_{I}+\mu_{I})}+\frac{\alpha \beta \left(1-r\right)S\left(t_{1}\right)}{\delta_{A}N}\\ & = & \left(1-m\left(t_{1}\right)\right)(\frac{\beta r}{\eta_{1}+\eta_{2}+\delta_{I}+\mu_{I}}+\frac{\alpha \beta (1-r)}{\delta_{A}})\\ & = & (1-\frac{1}{N}\int_{0}^{t_{1}}\phi \left(t_{1}-\eta \right)V^{\prime }\left(\eta \right)d\eta )(\frac{\beta r}{\eta_{1}+\eta_{2}+\delta_{I}+\mu_{I}}+\frac{\alpha \beta (1-r)}{\delta_{A}}). \end{array}$$ (10)

The above expression of the controlled reproduction number depends upon the vaccine efficacy *ϕ*(*η*) and the daily number of vaccinations *V*′(*η*). This expression of reproduction number suggests that a lower level of immunity at time *t*_1_ leads to larger value of $R_{c}$ and consequently a larger peak of epidemic.

## 3 Under-reporting in infected and in death: A reason for larger epidemic peak

Under-reporting in infected is an important aspect that can significantly influence the spread of epidemic. Here, by the term ***under-reporting in infected***, we mean a subclass of the symptomatic infected individuals where individuals do not move from the symptomatic infected compartment to the quarantine or hospitalization. The symptoms onset of an infected individual is mainly determined by the total viral load in the upper respiratory track of an infected individual. Asymptomatic individuals generally have lower viral loads [28–30] and consequently the disease transmission rate is also smaller for them. Whereas, a symptomatic infected has a higher viral load inside his/her body and consequently the transmission rate is higher for them. Thus under-reporting in the symptomatic infected compartment can play a crucial role.

Since, the asymptomatic individuals do not develop any infection related symptoms, they are not tested for the infection and their numbers remain unaccounted for in the official records. In contrast, the complete reporting of symptomatic individuals can be made compulsory but which is not the case for COVID-19 due to various reasons. One possible explanation for under-reporting lies in the individual-level use of testing kits. In this scenario, individuals who test positive may not be included in the reported data due to the lack of awareness of the individuals. Furthermore, symptomatic individuals, despite being conscious of their infectiousness, may not encourage reporting due to many reasons like, fear of social stigma and discrimination, government policies etc. These factors collectively contribute to the challenge of achieving complete reporting of symptomatic cases in the context of COVID-19. As a matter of fact, from the controlling perspectives, we consider under-reporting as an important factor which is defined as the proportion of symptomatic cases who were not reported to the surveillance authority [31]. To account for under-reporting in symptomatic compartment, we introduce a new parameter *u* ∈ (0, 1] representing a fraction of symptomatic cases that are reported. Accordingly, *I*_1_(*t*) and *I*_2_(*t*) represent the reported and under reported infected compartments which are two sub-compartments of the symptomatic compartment *I* in system (8). To be precise, *I*_1_ = *urσE* and *I*_2_ = (1 − *u*)*rσE*. In the presence of under-reporting, the growth equations for *I*_1_ and *I*_2_ compartments are given by:

$$\begin{array}{ccc} \frac{dI_{1}}{dt} & = & ur\sigma E-\left(\eta_{1}+\eta_{2}+\delta_{I}+\mu_{I}\right)I_{1} \end{array}$$ (11a)

$$\begin{array}{ccc} \frac{dI_{2}}{dt} & = & \left(1-u\right)r\sigma E-\left(\delta_{I}+\mu_{I}\right)I_{2}. \end{array}$$ (11b)

The complete model with *I*_1_ and *I*_2_ compartments is described in (16) at Appendix. (7.2) in S1 Appendix. In the complete model, the new parameter *α*_*u*_ is the relative transmission rate of the under-reported compartment and all other parameters are same as described earlier for the model (8).

### 3.1 Controlled reproduction number

Now, we find the controlled reproductive number for the under-reported system (see 16 in Sec. 7). Consider the equilibrium point (*S*(*t*_1_), 0, 0, 0, 0, 0, 0, 0, 0). Now,

$$\begin{array}{ccc} S(t_{1}) & = & N(1-m(t_{1}))=N-\int_{0}^{t_{1}}\phi \left(t_{1}-\eta \right)V^{\prime }\left(\eta \right)d\eta . \end{array}$$

We use the next generation matrix approach [27], to calculate the controlled reproduction number. Details of the calculations are given in 7.2.

The reproduction number at time *t* = *t*_1_ is the spectral radius of the matrix *FV*^−1^ and is given by:

$$\begin{array}{ccc} R_{c}^{1} & = & \frac{\beta S(t_{1})ur}{N(\eta_{1}+\eta_{2}+\delta_{I}+\mu_{I})}+\frac{\alpha_{u}\beta \left(1-u\right)rS\left(t_{1}\right)}{\delta_{I}+\mu_{I}}+\frac{\alpha \beta \left(1-r\right)S\left(t_{1}\right)}{\delta_{A}N}\\ & = & \left(1-m\left(t_{1}\right)\right)(\frac{\beta ur}{\eta_{1}+\eta_{2}+\delta_{I}+\mu_{I}}+\frac{\alpha_{u}\beta \left(1-u\right)r}{\delta_{I}+\mu_{I}}+\frac{\alpha \beta (1-r)}{\delta_{A}})\\ & = & (1-\frac{1}{N}\int_{0}^{t_{1}}\phi \left(t_{1}-\eta \right)V^{\prime }\left(\eta \right)d\eta )(\frac{\beta ur}{\eta_{1}+\eta_{2}+\delta_{I}+\mu_{I}}+\\ & & \frac{\alpha_{u}\beta \left(1-u\right)r}{\delta_{I}+\mu_{I}}+\frac{\alpha \beta (1-r)}{\delta_{A}}). \end{array}$$

**Fig 4:**
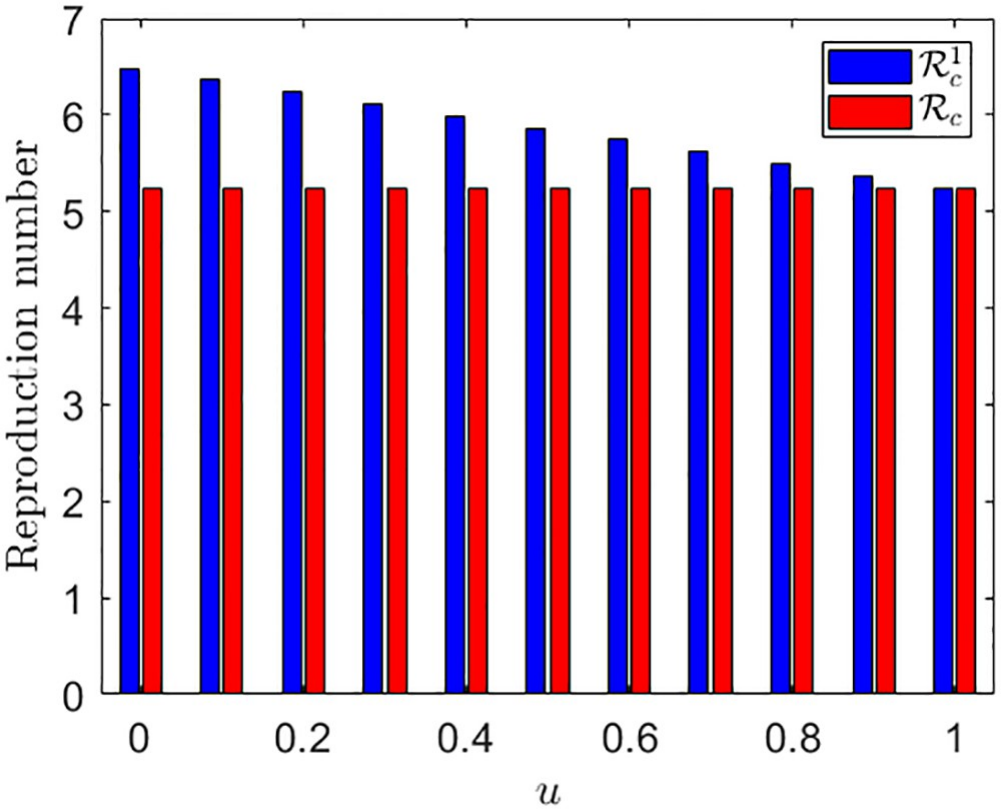
The red and blue color correspond to the controlled reproduction numbers with under-reporting and without under-reporting respectively. All other parameter values are same as in the green curves in Fig (17).

**Note:** If *u* = 1, then $R_{c}^{1}=R_{c}$, and for any *u* ∈ (0, 1), $R_{c}^{1}\geq R_{c}$. In Fig (4), a comparison between the controlled reproduction numbers with under-reporting and without under-reporting is shown. Note that *u* = 1 corresponds to the case of no under-reporting, and in that case both the reproduction numbers $R_{c}^{1}$ and $R_{c}$ are equal. As the value of *u* decreases, the reproduction number $R_{c}^{1}$ increases (blue curve).

### 3.2 Imitation dynamics of under-reporting in infected

In the context of COVID-19 in China, when the zero-COVID policy was relaxed, then there might be sudden significant changes in human behavior. Since, the govt. relaxed the restrictions and the BA.2 sub-variant of Omicron was comparatively less severe, people may be inclined towards under-reporting. In addition to that, the imitation dynamics can also play a crucial role in under-reporting. An individual who initially was inclined towards reporting, may change his/her mind towards under-reporting when he/she finds people around him/her who are inclined towards under-reporting, and vice-versa. Thus in more realistic scenario, the proportion of under-reporting can depend on the behaviors of individuals and one of the important behavior is the imitation.

Under-reporting can be considered as a type of game dynamics, similar to the imitation game. In the imitation game, individuals observe the behavior of their peers and adopt similar strategies in order to achieve a certain outcome. Similarly, in situations where reporting is required, individuals may observe the behavior of others and adopt similar strategies. The payoff regarding the imitation game dynamics, represents the benefit or cost associated with a particular action or strategy, and the payoffs are typically taken as negative for the following reasons:

i. Reporting carries some cost: Reporting often involves some monetary cost, as well as the time and effort required to be quarantined or hospitalized. This cost is typically reflected as a negative payoff.
ii. Some people may perceive quarantine, a consequence of reporting, as an unpleasant experience, which may also be reflected as a negative payoff.
iii. The negative consequences of under-reporting: Under-reporting can result in negative consequences, such as spreading the disease to others, severity of the infection and potentially even death. These negative consequences are typically reflected as negative payoffs.

Suppose, −*p*_*r*_ denotes the payoff related to reporting, where, *p*_*r*_ accumulates some monetary cost, as well as the time and effort required to be quarantined or hospitalized, and −*p*_*u*_ is the payoff related to under-reporting, where, *p*_*u*_ is the perceived risk of suffering serious conditions and sometimes death due to infection. These perceived risk (or cost) factors *p*_*r*_ and *p*_*u*_ typically lie between 0 and 1 because they are usually expressed as the probabilities or percentages, where, 0 represents no risk (or cost) and 1 represents highest possible risk (or cost). The net payoff gain ΔP is defined by

$$\Delta P=-p_{r}-\left(-p_{u}\right)=-p_{r}+p_{u}.$$

Then the human behavior based imitation dynamics of *u* is described by (following the derivation by Bauch [32]):

$$\begin{array}{c} \frac{du}{dt}=c\Delta Pu\left(1-u\right)=c\left(-p_{r}+p_{u}\right)u\left(1-u\right), \end{array}$$ (12)

where, *c* is the imitation rate. It is important to mention here that the initial condition for *u* lies between 0 and 1, i.e., 0 ≤ *u*(0) ≤ 1.

#### Constant rate of under-reporting in infected

Note that, if the net payoff gain is zero, i.e., ΔP=0, then $\frac{du}{dt}=0,$ which is the situation of constant rate of under-reporting.

## 4 Parameter estimation using physics informed neural networks

One of the challenges in using compartmental models with associated differential equation system to describe the dynamics is the *estimation of parameters* for given data. Usually, parameters may be estimated from observed patterns in the data, but transmission rates often have to be computed using heuristic algorithms that are computationally or statistically motivated. In the recent years, there have been new approaches with *machine learning* to discover parameters [17]. One of these approaches includes Artificial Neural Networks (ANN) which are motivated by the human neural system where each neuron is represented with a node, signals are inputs, and synapse is represented as a function evaluation. Each neuron is connected to different neurons in multiple layers, which leads to decrease the error using non-linear approximations. ANN have been used in regression and classification tasks in the last few years. By adding the physics behind the given problem, i.e., the system of equations, a new approach was proposed called Physics-Informed Neural Networks or PINNs [18]. These neural networks encode model equations, like Partial Differential Equations (PDEs), as a component of the neural network itself [33].

**Fig 5:**
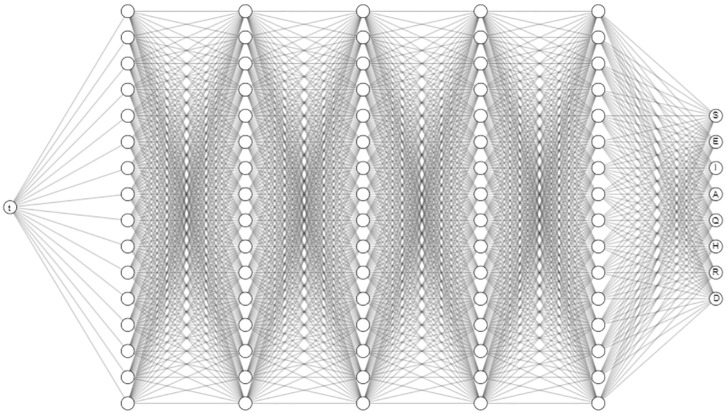
Neural Network architecture for DINNs applied to system (8a)-(8h).

This approach aims to solve two main classes of problems: *data-driven solution* and *data-driven discovery* of differential equations. It is a fast and mesh-free method. However, it is still in early development but is starting to be applied to infectious diseases. Recently, Disease Informed Neural Networks (DINNs) was proposed to leverage the hidden physics of infectious diseases and infer the latent quantities of interest by approximating them using PINNs [19]. The neural network architecture for system (8a) and (8b) is showed in Fig 5.

For the function *t* → (*S*, *E*, *I*, *A*, *Q*, *H*, *R*, *D*), the system (8a)-(8h) corresponds to the following residuals:

$$\begin{array}{cc} L_{S} & =\frac{dS}{dt}+\frac{\beta S}{N}\left(I+\alpha A\right)+\int_{0}^{t}\phi^{\prime }\left(t-\eta \right)V^{\prime }\left(\eta \right)d\eta ,\\ L_{E} & =\frac{dE}{dt}-\frac{\beta S}{N}\left(I+\alpha A\right)+\sigma E,\\ L_{I} & =\frac{dI}{dt}-r\sigma E+\left(\eta_{1}+\eta_{2}+\delta_{I}+\mu_{I}\right)I,\\ L_{A} & =\frac{dA}{dt}-\left(1-r\right)\sigma E+\delta_{A}A,\\ L_{Q} & =\frac{dQ}{dt}-\eta_{1}I+\left(\xi +\delta_{Q}\right)Q,\\ L_{H} & =\frac{dH}{dt}-\eta_{2}I-\xi Q+\left(\delta_{H}+\mu_{H}\right)H,\\ L_{R} & =\frac{dR}{dt}-\delta_{I}I-\delta_{A}A-\delta_{H}H-\delta_{Q}Q,\\ L_{D} & =\frac{dD}{dt}-\mu_{I}I-\mu_{H}H. \end{array}$$

This neural network is trained using temporal data, which estimate the best values of the model parameters without much prior information. Let the unknown solution be a vector of eight components such that

$$u\left(t;\lambda \right)={\left[S(t;\lambda ),E(t;\lambda ),I(t;\lambda ),A(t;\lambda ),Q(t;\lambda ),H(t;\lambda ),R(t;\lambda ),D(t;\lambda )\right]}^{⊤}$$

where λ are the parameters related to the disease dynamics, and a known initial condition *u*(0).

The training data has been discretized, as time {*t*^*j*^} and solution {*u*^*j*^}, where *j* = 0, 1, …, *N*_data_ such that *t*^0^ = 0 corresponds to an initial condition. The goal then is to train a neural network with parameters (λ^⊤^, *θ*^⊤^)^⊤^ where *θ* is a concatenation of weights and biases for each artificial neuron. The optimization process obtains a vector ${\left({\hat{\lambda }}^{⊤},{\hat{\theta }}^{⊤}\right)}^{⊤}$ and an approximation ${\hat{u}}^{j}\left(\hat{\lambda },\hat{\theta }\right)$ using the following loss function

$$L\left(\hat{\lambda },\hat{\theta }\right)=\omega_{\text{ode}}L_{\text{ode}}\left(\hat{\lambda },\hat{\theta }\right)+\omega_{\text{ic}}L_{\text{ic}}\left(\hat{\lambda },\hat{\theta }\right)+\omega_{\text{data}}L_{\text{data}}\left(\hat{\lambda },\hat{\theta }\right)$$

where *ω*_ode_, *ω*_ic_ and *ω*_data_ are the loss weights of the loss functions of the system of differential equations, initial conditions and training data, respectively. While the overall loss function is decomposed in other three parts, the loss function from the differential equation may be expressed as:

$$\begin{array}{cc} L_{\text{ode}}\left(\hat{\lambda },\hat{\theta }\right) & =L_{S}\left(\hat{\lambda },\hat{\theta }\right)+L_{E}\left(\hat{\lambda },\hat{\theta }\right)+L_{I}\left(\hat{\lambda },\hat{\theta }\right)+L_{A}\left(\hat{\lambda },\hat{\theta }\right)\\ & \;+L_{Q}\left(\hat{\lambda },\hat{\theta }\right)+L_{H}\left(\hat{\lambda },\hat{\theta }\right)+L_{R}\left(\hat{\lambda },\hat{\theta }\right)+L_{D}\left(\hat{\lambda },\hat{\theta }\right). \end{array}$$

The loss function corresponding to the data maybe expressed as:

$$L_{\text{data}}\left(\hat{\lambda },\hat{\theta }\right)=\sum_{i=1}^{8}\frac{1}{N_{\text{data}}}\sum_{j=1}^{N_{\text{data}}}{\left(u_{i}^{j}-{\hat{u}}_{i}^{j}\left(\hat{\lambda },\hat{\theta }\right)\right)}^{2}.$$

The loss function corresponding to the initial condition may be expressed as:

$$L_{\text{ic}}\left(\hat{\lambda },\hat{\theta }\right)=\sum_{i=1}^{8}{\left(u_{i}^{0}-{\hat{u}}_{i}^{0}\left(\hat{\lambda },\hat{\theta }\right)\right)}^{2}$$

where *u*_*i*_ for *i* = 1, …, 8 corresponds to solution to each compartment in system (8a)–(8h), for example, *u*_1_ corresponds to *S*, *u*_2_ corresponds to *E* and so on until *u*_8_ corresponds to *D*. Algorithm 1 shows how to estimate *u*(*t*; λ) and the parameters λ.

**Algorithm 1**: DINN algorithm.

**Input:** Training Data {*t^j^*}, {*u^j^*} where *j* = 0, 1, …, *N_data_*

**Output:** $\hat{u}$ and $\hat{\lambda }$

1 Initialize ${\hat{\lambda }}_{0}$ and ${\hat{\theta }}_{0}$

2 Define time interval where the solution will be found.

3 Define loss function $L(\hat{\lambda },\hat{\theta })$, related to residual errors, initial conditions and training data.

4 Create a fully connected neural network with 1 neuron in the input layer and 8 neuron in the output layer (one per compartment) and such that it normalize the input data.

5 Choose optimization hyper-parameters (e.g. Adam optimizer, learning rate and loss weights).

6 **for**
iter = 1, …, max_iter
**do**

7  Compute total loss $L({\hat{\lambda }}_{\text{iter}-1},{\hat{\theta }}_{\text{iter}-1})$, in particular is necessary to use auto-differentiation for ODE residuals.

8  Train neural network with optimizer algorithm and update ${\hat{\theta }}_{\text{iter}-1}$ to ${\hat{\theta }}_{\text{iter}}$.

9  Get approximation ${\hat{u}}_{\text{iter}}$ and ${\hat{\lambda }}_{\text{iter}}$.

10 Return ${\hat{u}}_{\text{max}\_\text{iter}}$ and ${\hat{\lambda }}_{\text{max}\_\text{iter}}$.

For our computations, we first estimated,

$$\begin{array}{c} \frac{dm}{dt}=\frac{1}{N}\int_{0}^{t}\phi^{\prime }\left(t-\eta \right)V^{\prime }\left(\eta \right)d\eta . \end{array}$$ (13)

**Fig 6:**
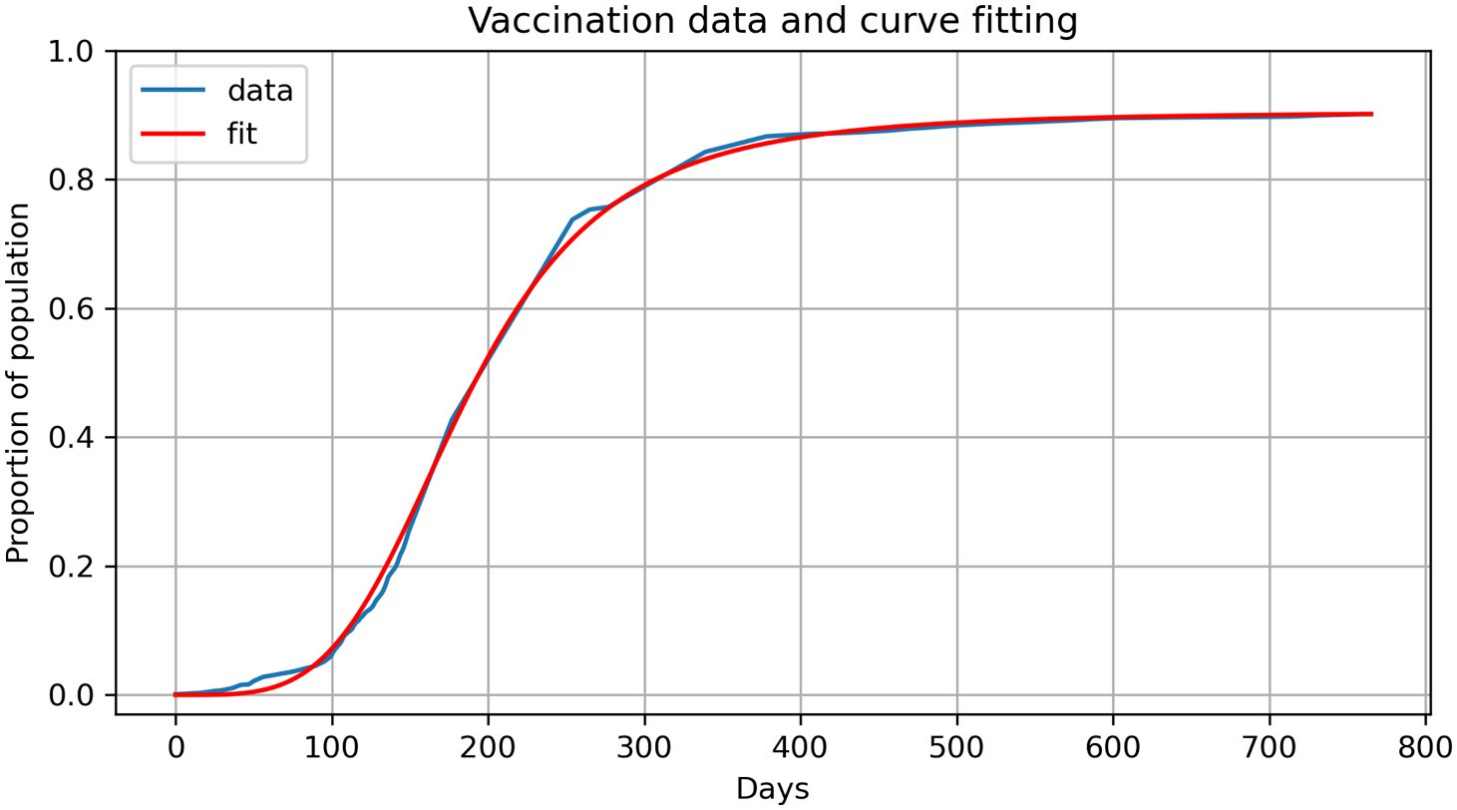
Proportion of population vaccinated and curve fitting.

Specifically, we first estimated the best-fit sigmoid function for *V*(*η*) using the real-data for vaccinations from Jan 2021 to Jan 2023 [10] given by:

$$\tilde{V}\left(t\right)=v_{1}(1-\frac{v_{2}}{v_{2}+t^{4}})$$

where *v*_1_ = 0.904733394 and *v*_2_ = 1.16081503 × 10^9^. Fig 6 shows the fit for this function in comparison to the vaccination data and we use the derivative of the function in Eq (13).

We then calculated *ϕ*(*t*) using specific values of (*b*, *c*) = (0.231, 0.0084), for a particular choice of the vaccine efficacy function *ϕ*(*t*) by the acquisition-fading kernel, as given in Eq (14). From the explicit form that we obtain for *ϕ*(*t*), we compute *ϕ*′(*t* − *η*) to be used in Eq (13).

**Fig 7:**
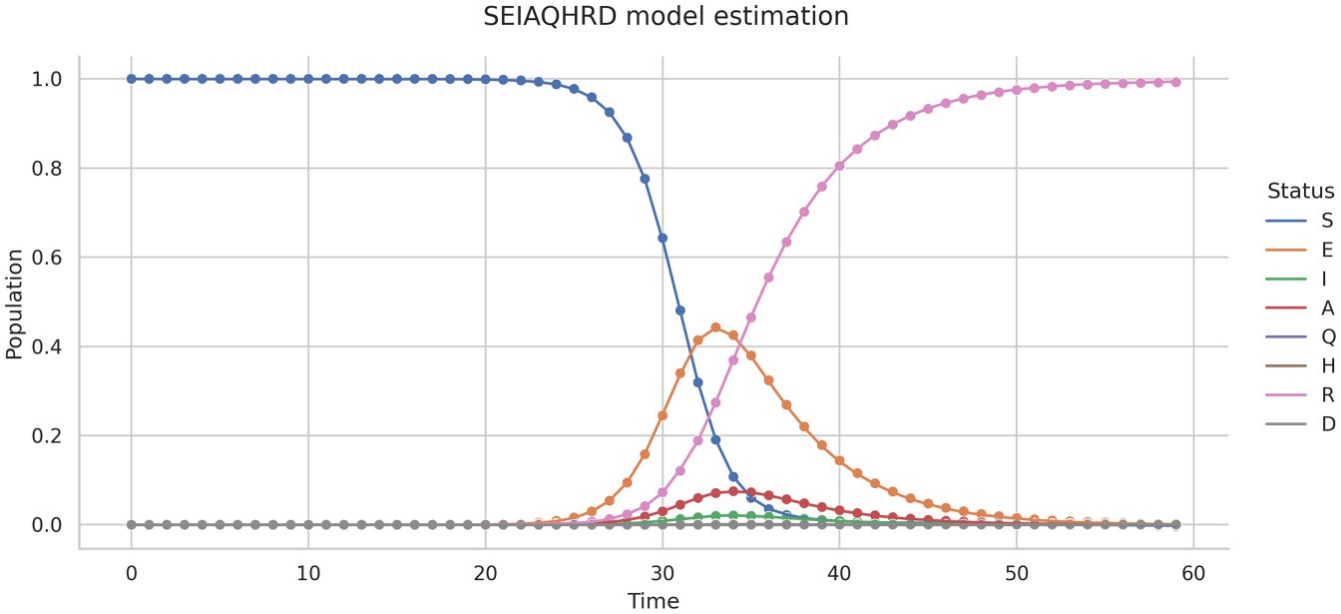
System (8a)-(8h) estimation using synthetic data, dots correspond to training data and lines correspond to DINNs prediction.

Once the integral in Eq (13) is numerically computed, this is explicitly used in solving the system (8a)-(8h) using the DINNs approach explained earlier. The neural network was able to learn the dynamics of the system (8a)-(8h) (see Fig 7).

**Fig 8:**
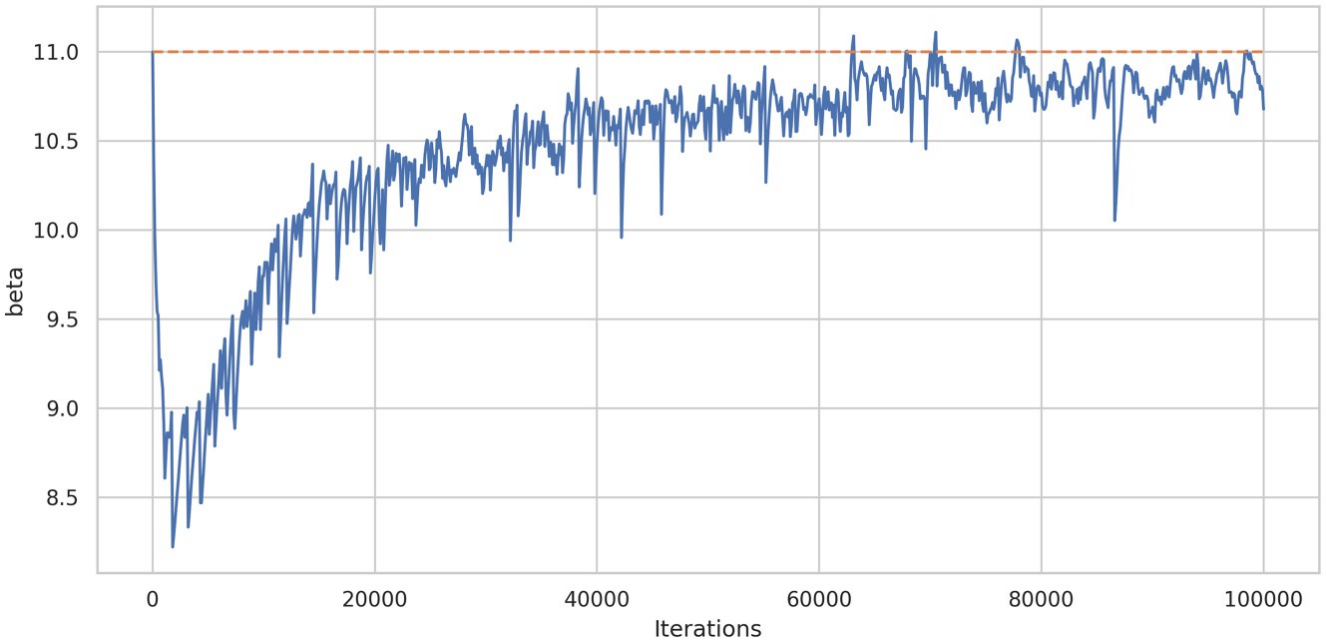
Learning history of parameter estimation (only *β*) of system (8) using DINNs.

As a first experiment, we fixed every parameter but we applied the framework only for estimating *β*, the rest of the parameters were fixed. Fig 8 shows the learning history of the DINNs framework. For this experiment in particular we fixed *β* = 11 and the approximation was $\hat{\beta }=10.6782$, which means a relative error equals to 0.02924.

**Fig 9:**
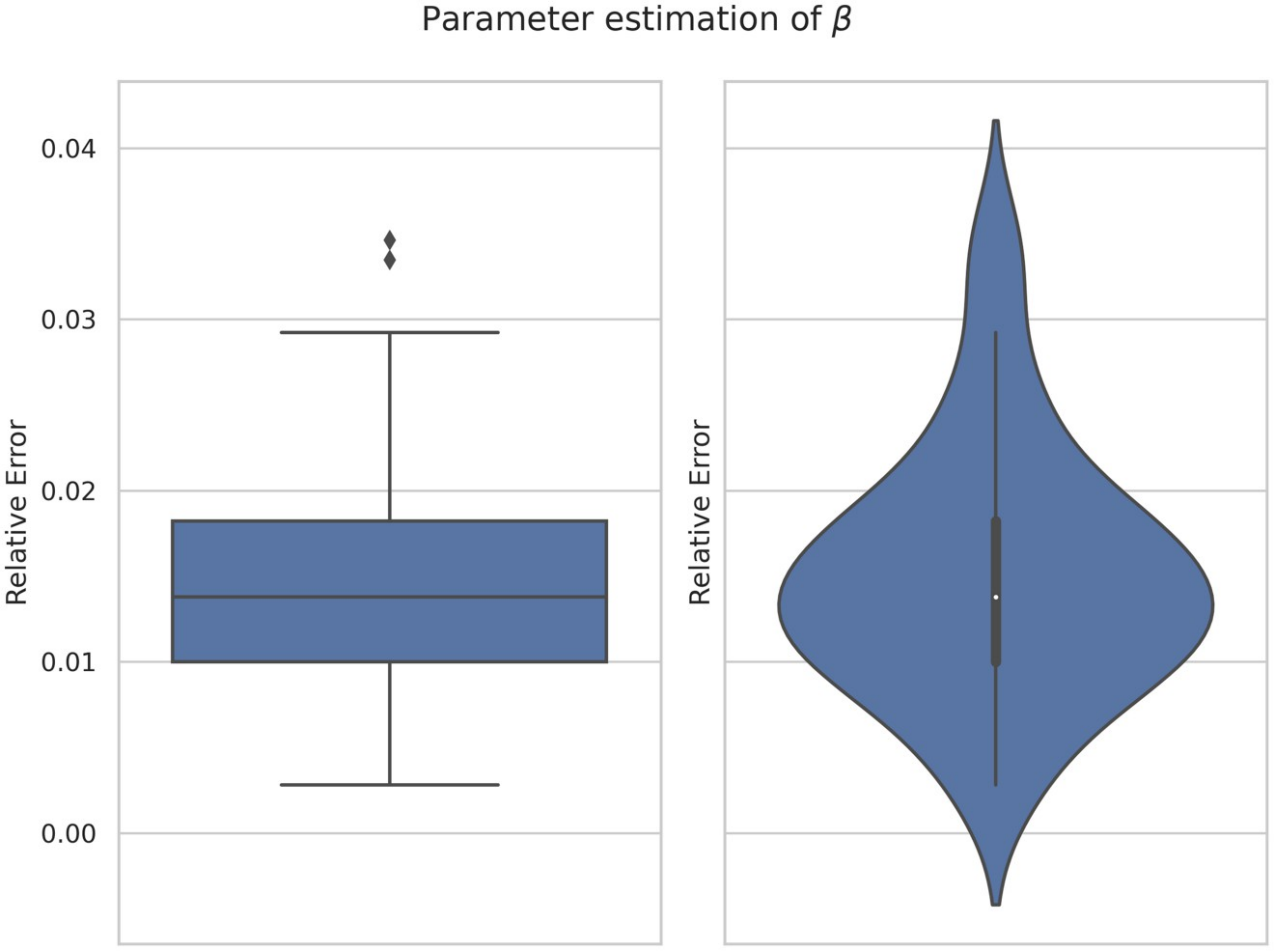
Box-plot (left) and violin-plot (right) of the relative error of *β* estimation corresponding to 40 simulations.

**Fig 10:**
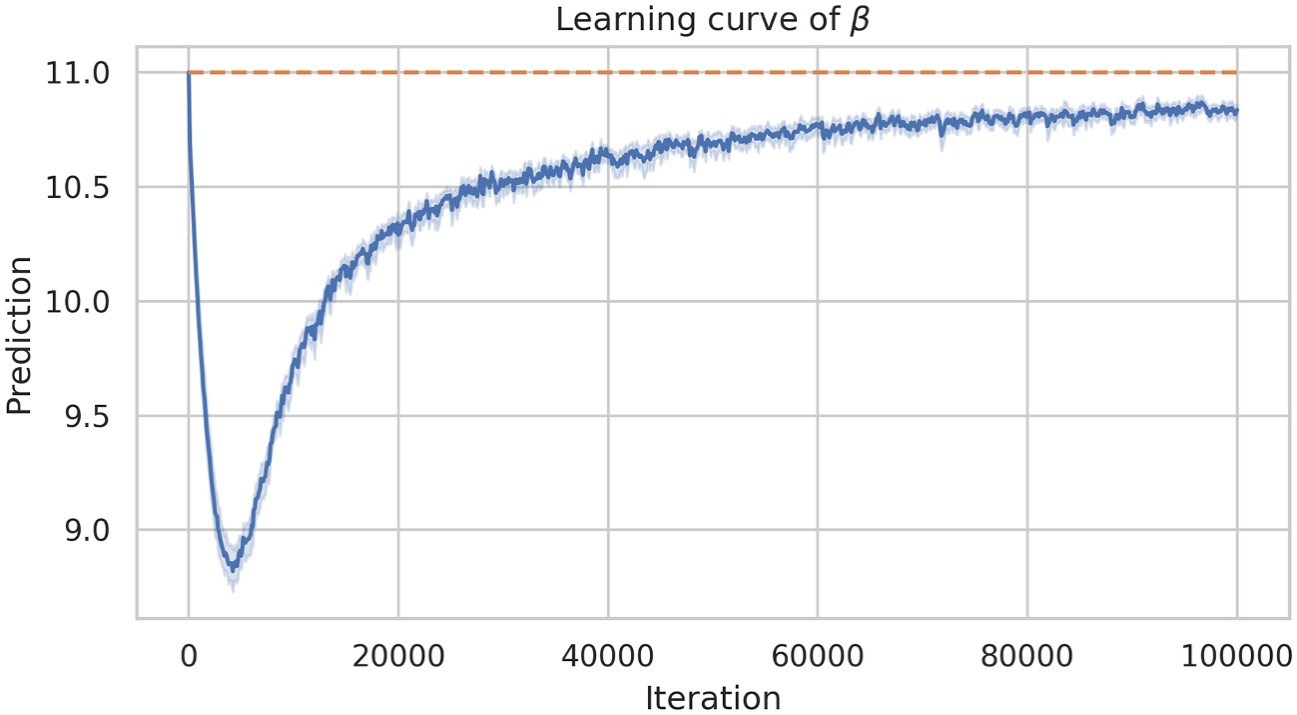
Mean learning history and its 95% confident interval of *β* estimation corresponding to 40 simulations.

In order to study the robustness of the framework we have run the model 40 times. Fig 9 shows there is no a large variation of the error. Similarly, Fig 10 shows the average learning history of *β* with a 95% confidence interval corresponding to the 40 simulations. Since this confidence interval is small we can support the robustness of this model as well.

### 4.1 Multi-parameter estimation

The predictive reliability of any inverse algorithm depends on its ability to apply the technique to estimate multiple parameters. In traditional non-linear least-squares approaches, it is well known that the approximations start to become worse when such optimization techniques try to estimate more parameters. Here we apply the DINNs approach described in this article to estimate more parameters next.

**Fig 11:**
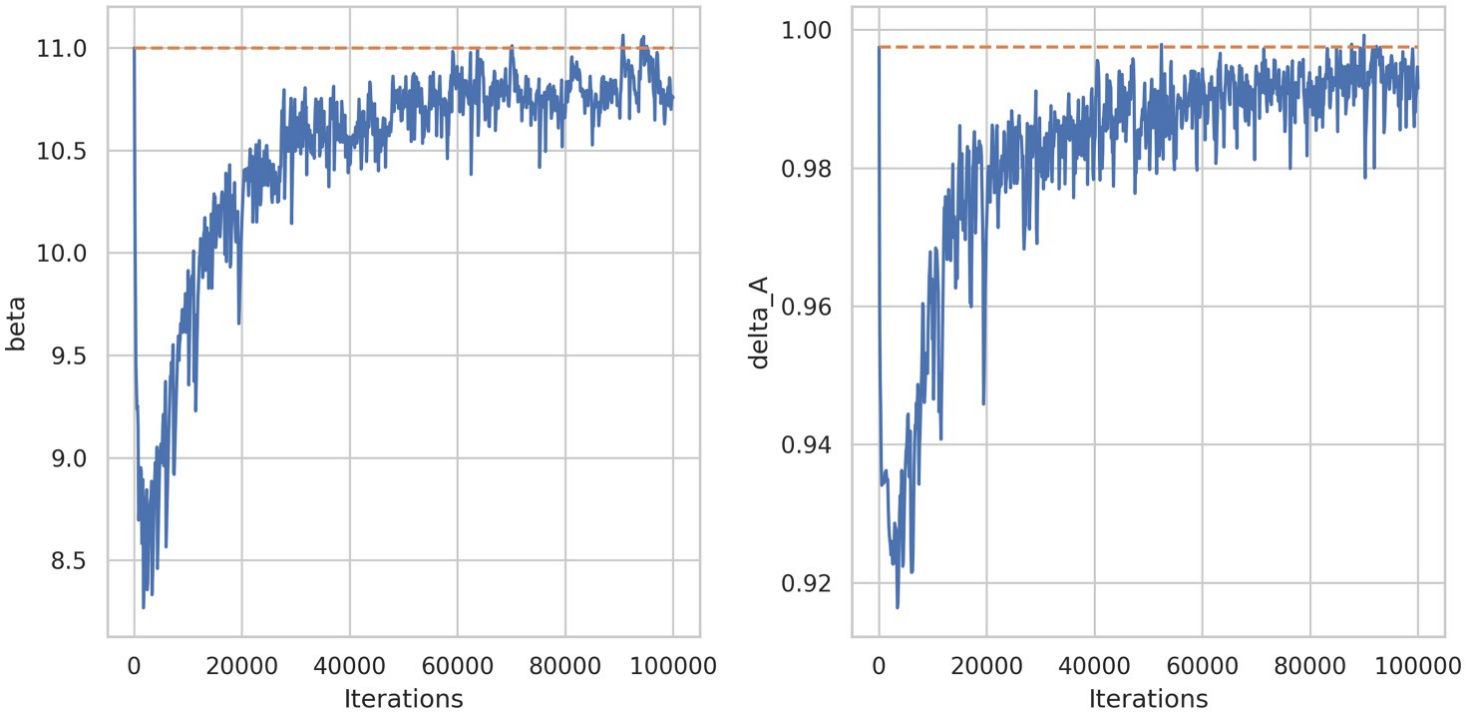
Learning history of parameter estimation (*β* and *δ*_*A*_) of system (8) using DINNs.

Specifically, we try to estimate two parameters, namely, *β* and *δ*_*A*_, where data prediction is similar to the first experiment. Fig 11 shows the learning history of these two parameters and Table 2 summarizes predictions and relative errors.

**Table 2:** Parameter predictions and relative errors for system (8) using DINNs.

	Real	Predicted	Rel. Error
*β*	11	10.7587	0.02193
*δ*_*A*_	0.9975	0.9915	0.00596

**Fig 12:**
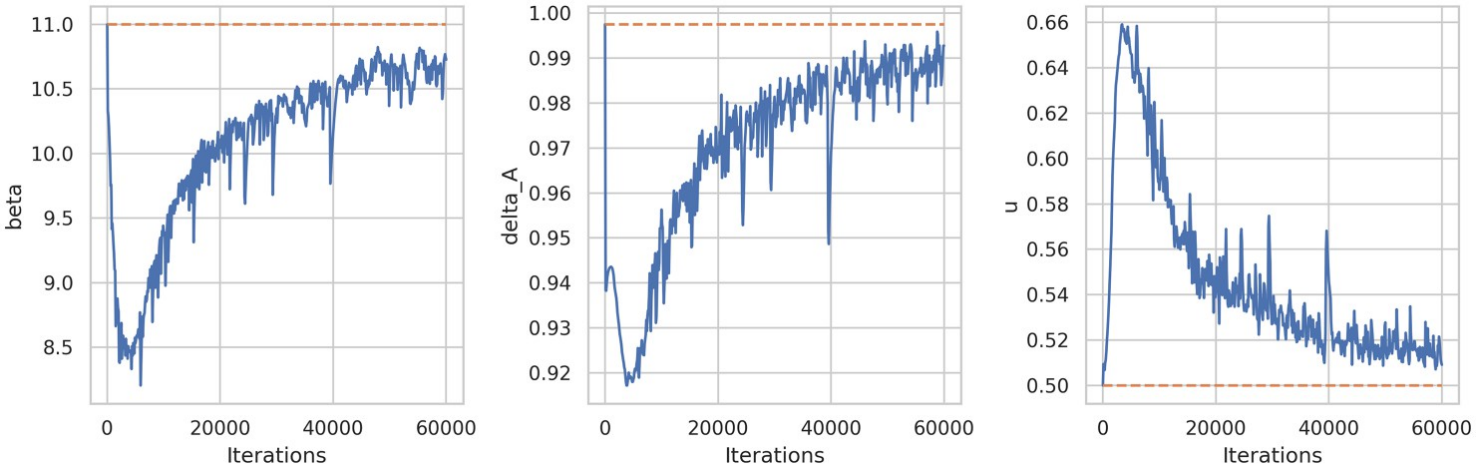
Learning history of parameter estimation (*β*, *δ*_*A*_ and *u*) of system (16) using DINNs.

The next experiment considered the under-reported scenario corresponding to system (16) where we estimated *β*, *δ*_*A*_ and *u*. Fig 12 shows the learning history of these two parameters and Table 3 summarizes predictions and relative errors.

**Table 3:** Parameter predictions and relative errors for system (16) using DINNs.

	Real	Predicted	Rel. Error
*β*	11	10.7269	0.02481
*δ*_*A*_	0.9975	0.9927	0.00472
*u*	0.5	0.5091	0.01825

**Fig 13:**
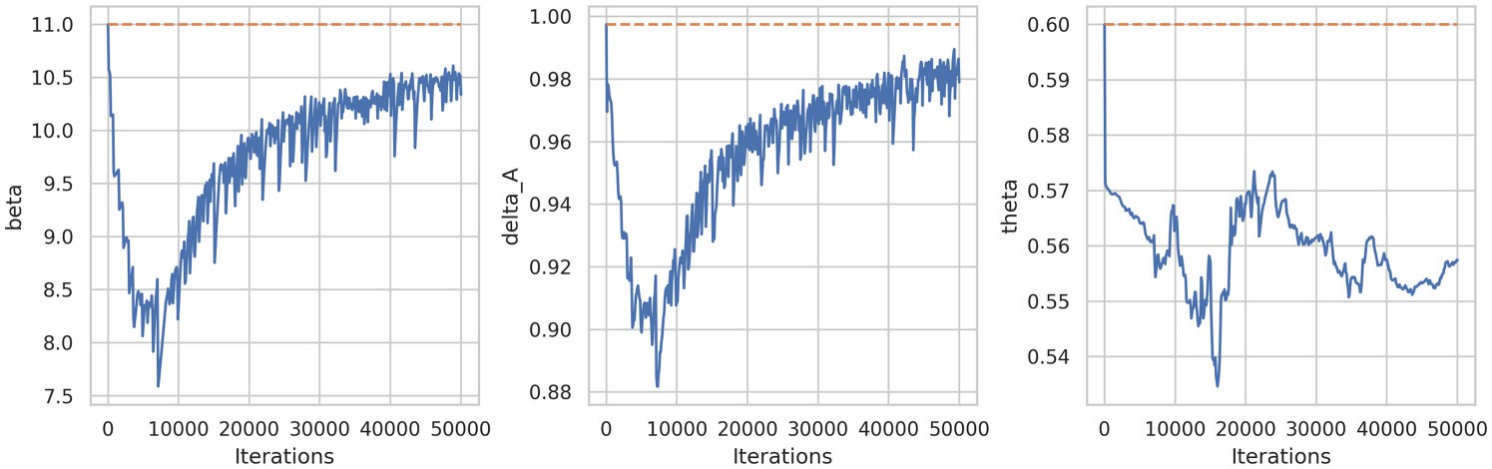
Learning history of parameter estimation (*β*, *δ*_*A*_ and *θ*) of system (16) and Eq (15) using DINNs.

Next, we used the same system (16) but now considering *u* as a function, as defined in Eq (15) and *p*_*u*_(*θ*) = *ρ*(1 − *θ*). We ran a similar experiment to learn the parameters *β*, *δ*_*A*_ and *θ*. See Fig (13) for parameter prediction history and Table 4 summarize parameters estimation with its relative errors.

**Table 4:** Parameter predictions and relative errors for system (15) and (16) using DINNs.

	Real	Predicted	Rel. Error
*β*	11	10.33896916	0.060093713
*δ*_*A*_	0.9975	0.979012692	0.018533643
*θ*	0.6	0.557463108	0.070894819

### 4.2 Influence of noise

**Fig 14:**
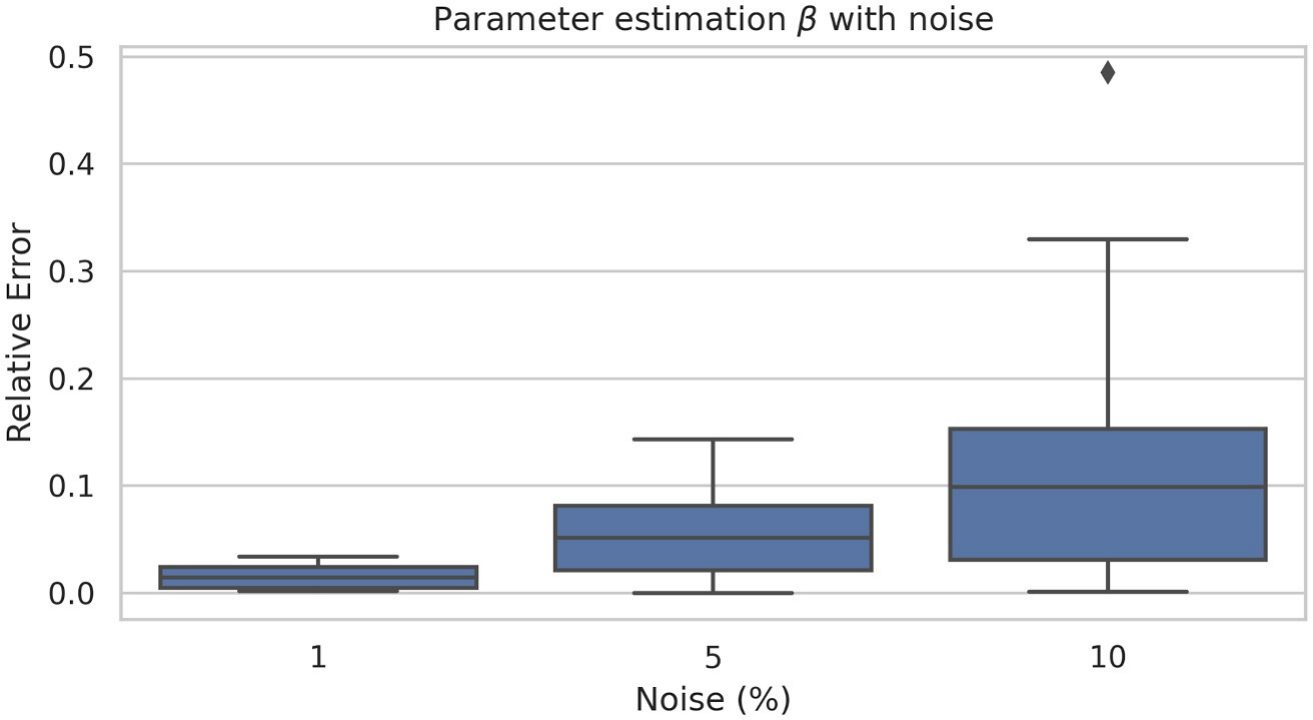
Box-plot of the relative error of *β* estimation corresponding to 30 simulations of each noise level (1%, 5% and 10%).

**Fig 15:**
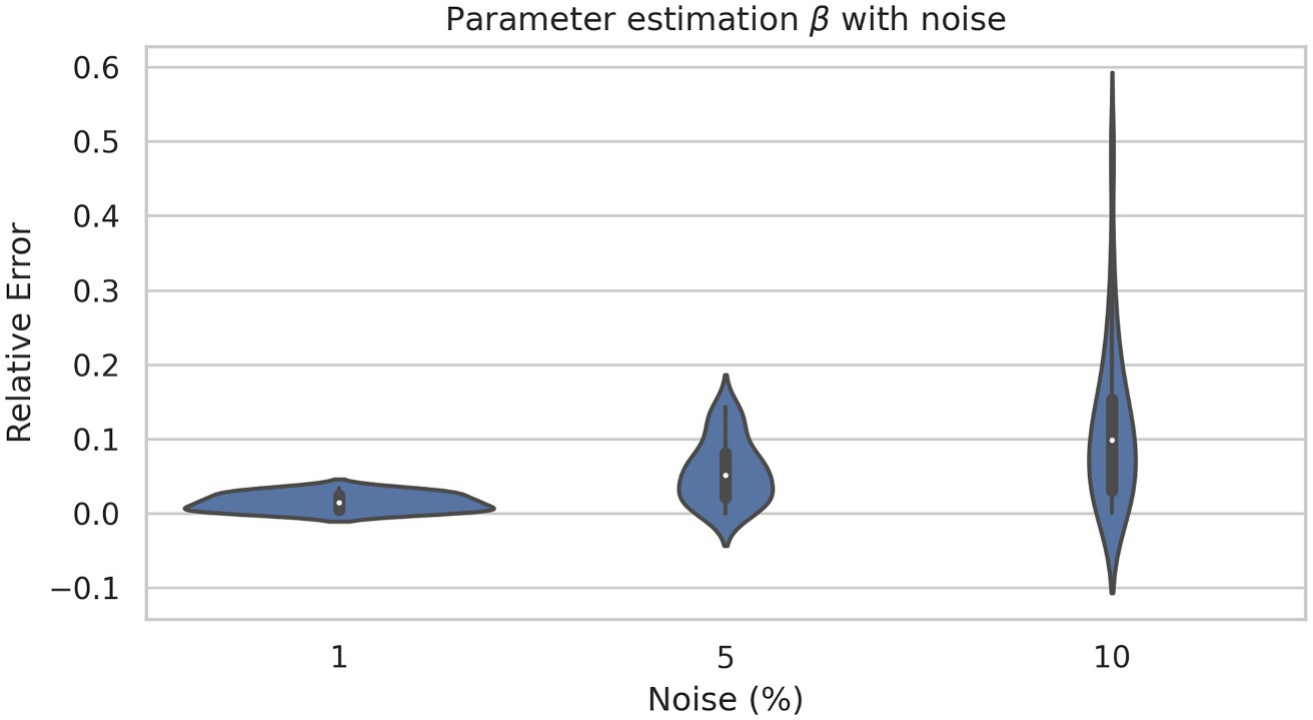
Violin-plot of the relative error of *β* estimation corresponding to 30 simulations of each noise level (1%, 5% and 10%).

**Fig 16:**
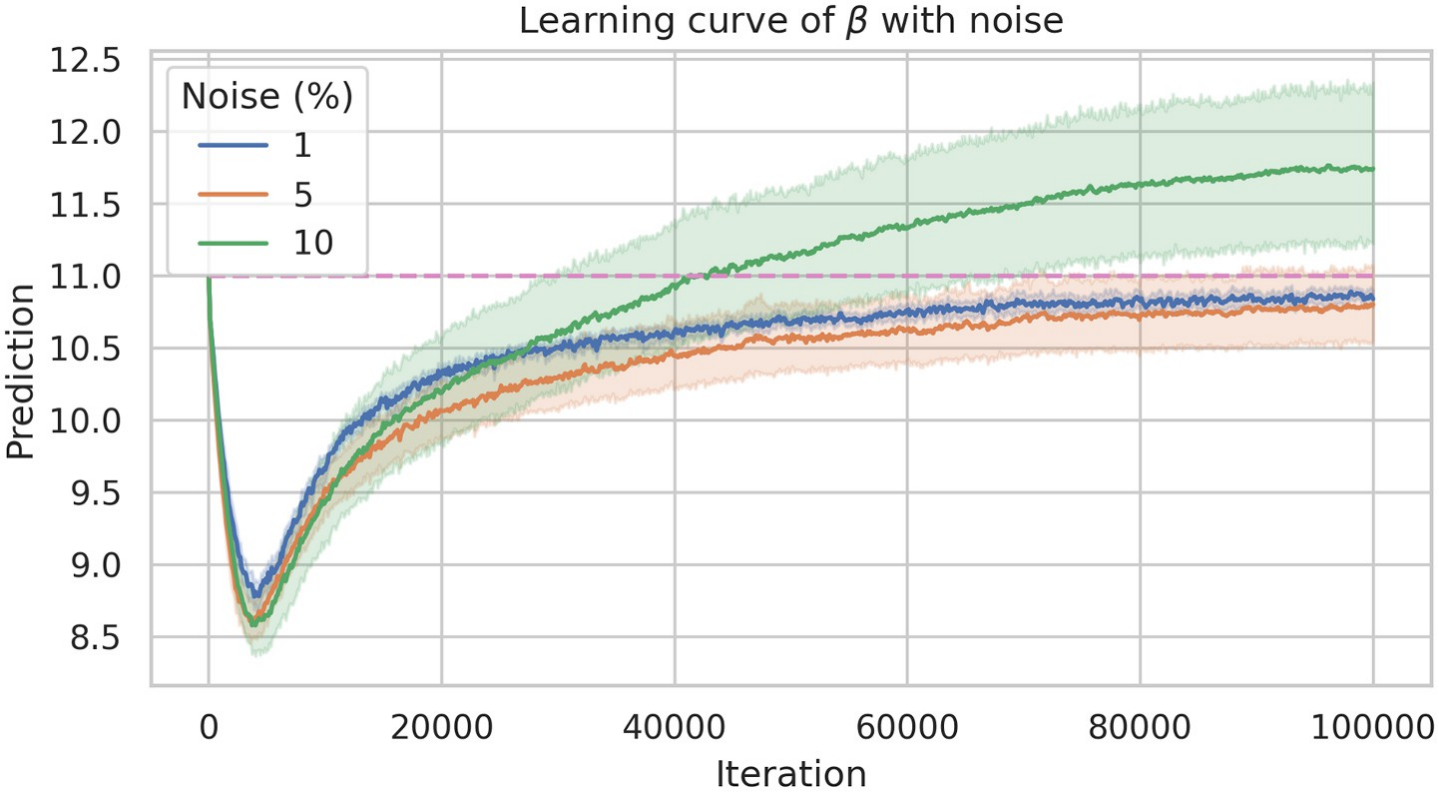
Mean learning history and its 95% confident interval of *β* estimation corresponding to 30 simulations of each noise level (1%, 5% and 10%).

To measure the robustness of our approach for the estimation approach to system (8), we perturb the data with different levels of noise (1%, 5% and 10%) on each compartment and then estimate *β*. Similar to the first estimation (single parameter) for studying robustness of the framework, we ran 30 simulations of each noise level. Figs 14 and 15 are box-plots and violin-plots of each noise level, showing what would be expected, this is, to increasing the noise results also increase the relative error and its spread as well. The history learning (see Fig 16) shows the same behavior, more noise increase the error and the confidence interval, but it is proportional to the noise level added to the training data.

### 4.3 Influence of missing data

Another measurement of robustness of an optimization technique is how the method predicts solutions when data is limited. In order to simulate it, we removed randomly 10%, 25% and 50% data points for the estimation of *β* = 11 in the system of Eq (8). Table 5 shows that even with increasing amount of missing data, the neural network method proposed herein, performs very well.

**Table 5:** *β* = 11 estimation and relative errors for system (8) using DINNs with different percentage of missing data.

Noise	Predicted	Rel. Error
10%	10.416714	0.053026
25%	10.738632	0.023761
50%	11.138561	0.012596

## 5 Results and findings

### 5.1 Effect of vaccine efficacy on epidemic progression

In this subsection, we study the effect of the vaccine efficacy on the disease progression with the help of numerical simulation. Here, we set *t* = 0 at the time when the vaccination started i.e., 15 December, 2020 for China, and *t*_1_ is 1st November, 2022 (because the outbreak started around the first week of November 2022, as per Worldometer data). The plot of *V*(*t*) for real data of cumulative vaccination in China starting from 15 December, 2020 to 23 December, 2022 is shown in Fig 1.

#### Effect of *ϕ*(*η*) on $R_{c}$

**Fig 17:**
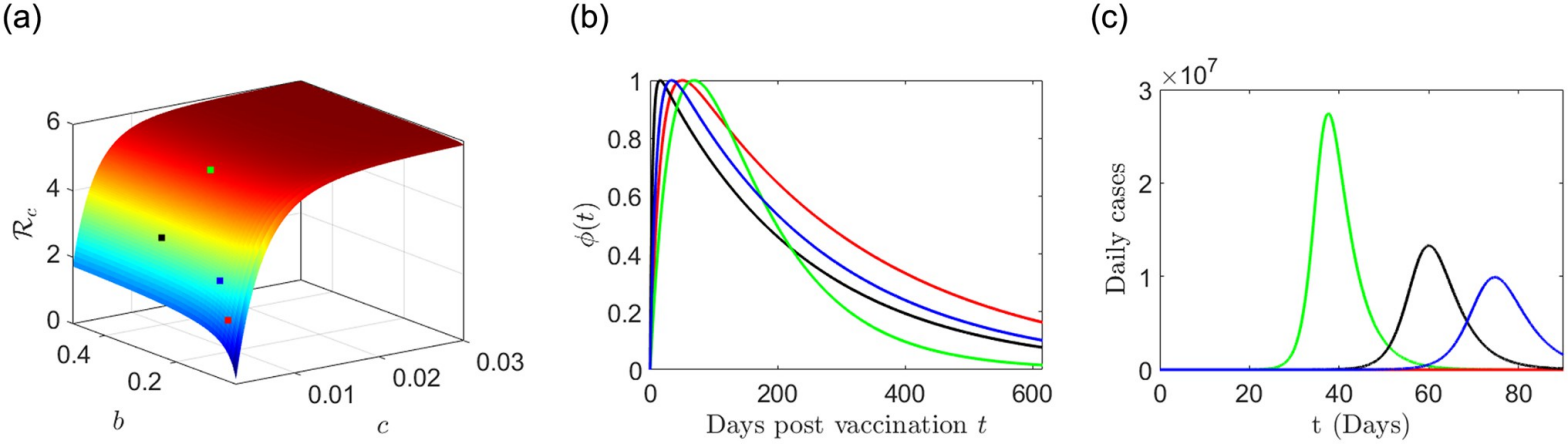
(a) Plot of the controlled reproduction number $R_{c}$ as a function of the fading rate *b* and the acquisition rate *c* as described in the formula (14); (b) Plot of the acquisition-fading kernel type vaccine efficacy function *ϕ*(*t*) = *A*(*e*^−*bt*^ − *e^−ct^*) (see, formula (14)) corresponding to different choice of *b* and *c* as follows: (*b*, *c*) = (0.231, 0.0084) (green), (*b*, *c*) = (0.061, 0.0033) (red), (*b*, *c*) = (0.271, 0.0043) (black), (*b*, *c*) = (0.1, 0.004) (blue) and the corresponding values of $R_{c}$ are indicated by the dots of corresponding color in the panel (a); (c) The number of daily cases for different choice of (*b*, *c*) are shown by corresponding colors.

Here, we study the effect of vaccine efficacy on the controlled reproduction number $R_{c}$. Vaccine efficacy begins to rise shortly after vaccination, reaching its peak after a few weeks (or months), and then it gradually declines over time-post-vaccination, due to waning of immunity [34]. To represent this pattern of vaccine efficacy, we consider a particular choice of the vaccine efficacy function by the acquisition-fading kernel, given by

$$\begin{array}{c} \phi \left(t\right)=A\left(e^{-bt}-e^{-ct}\right), \end{array}$$ (14)

where, *b* is the fading rate, *c* is the acquisition rate, *b* ≠ *c*, and *A* = 1/((*c*/*b*)^(*b*/(*b*−*c*))^ − (*c*/*b*)^(*c*/(*b*−*c*))^). For this particular choice of *ϕ*, in Fig 17a, we plot the controlled reproduction number $R_{c}$ as a function of *b* and *c*. From Fig 17, we observe that $R_{c}$ significantly depends upon the fading and acquisition parameters *b* and *c* respectively.

#### Note

The value of $R_{c}$ is significantly small in case of smaller values of both the fading and acquisition parameters *b* and *c* respectively. Thus, a vaccine efficacy function with slow acquisition and slow decaying nature, can effectively control the epidemic progression. If the vaccine efficacy rapidly decays (as in the green curve in Fig 17(b)), then the $R_{c}$ is very high (around 5.25 for the green curve), whereas, if the vaccine efficacy decays slowly (as in the red curve in Fig 17(b)), then the $R_{c}$ is significantly smaller (around 1.42 for the red curve).

#### Effect of *ϕ*(*η*) on daily cases

To numerically investigate the effect of vaccine efficacy and its duration on the daily number of cases of the infection, we numerically simulate the model (8) with parameter values given in Table 1, along with the efficacy function defined in (14). The vaccination data *V*(*t*) is shown in Fig 1. The initial conditions are: *S*(*t*_1_) = *N* − 1, *E*(*t*_1_) = 0, *I*(*t*_1_) = 1, *A*(*t*_1_) = 0, *Q*(*t*_1_) = 0, *H*(*t*_1_) = 0, *R*(*t*_1_) = 0 and *D*(*t*_1_) = 0, where *t*_1_ is 1st November, 2022 (as the outbreak started around the first week of November 2022, as per Worldometer data). We assumed the value of *β* = 11 to get the daily cases in the order of 10^7^. We simulate the model (8) with the vaccine efficacy function *ϕ*(*η*) (as in (14)) for the set of values of the acquisition and rate parameters as follows: (*b*, *c*) = (0.231, 0.0084), (0.061, 0.0033), (0.271, 0.0043), and (0.1, 0.004), corresponding to the green, red, black and blue curves, respectively, in Fig 17a and 17b. The simulation results for the daily cases are shown in Fig 17c by respective colors in Fig 17a and 17b. We observe that there is no outbreak corresponding to the red curves, whereas, the green curves produce a very large peak of the outbreak. Thus, although the number of vaccinated individuals are same, the less efficacy of the vaccine (i.e., the efficacy is decaying quickly) can lead to significantly different type of epidemic progression. In other words, the less effectiveness of the vaccine may be one of the reasonable factor to have faster epidemic growth and a large number of daily infected cases.

### 5.2 Under-reporting in death

Based upon the information available in various news media, there were under-reporting in death in China [12, 13]. Although, the under-reporting in death has no direct impact on the disease transmission, but it can indirectly influence the human behavior. If people receive suppressed data of death, then based upon that suppressed data people may decide that the severity and mortality of the disease is less, and this decision has an impact on −*p*_*u*_ (the payoff related to under-reporting of infected). To capture this implicit influence of under-reporting in death on the epidemic progression, we introduce another parameter *θ* ∈ [0, 1], which represents the proportion of under-reporting in death. Since the perceived payoff related to under-reporting of infected depends upon the information about the morbidity due to infection, we can assume that the payoff related to under-reporting of infected is a function of *θ*, i.e., −*p*_*u*_(*θ*). Then the Eq (12) becomes:

$$\begin{array}{c} \frac{du}{dt}=c\left(-p_{r}+p_{u}\left(\theta \right)\right)u\left(1-u\right). \end{array}$$ (15)

In a particular we can choose

$$p_{u}\left(\theta \right)=\rho \left(1-\theta \right),$$

where, *θ* is the proportion of death that is suppressed, and consequently, the proportion of reported information of death is (1 − *θ*). Just to explain, if *θ* = 1, which means all the deaths are under reported, then people may receive the information that the disease related death rate is zero, then,

$$p_{u}\left(\theta \right)=\rho \left(1-\theta \right)=0,$$

which implies from (15),

$$\begin{array}{ccc} \frac{du}{dt} & = & c\left(-p_{r}+p_{u}\right)u\left(1-u\right)\\ & = & -cp_{r}u\left(1-u\right). \end{array}$$

This shows *u* is decreasing, which signifies the fact that people are becoming more inclined due to under-reporting. Thus under-reporting in death as well as in infected can influence the epidemic progression.

We explain the implicit effect of under-reporting in death in the decision making of individuals which has an impact on determining the size of the epidemic peak. We simulate system (15) and (16) for three scenarios as follows:

i. Scenario-I: *θ* = 0.05, which means that only 5% data of death are under-reported or suppressed.
ii. Scenario-II: *θ* = 0.6, which means that 60% data of death are under-reported or suppressed.
iii. Scenario-III: *θ* = 0.95, which means that 95% data of death are under-reported or suppressed.

**Fig 18:**
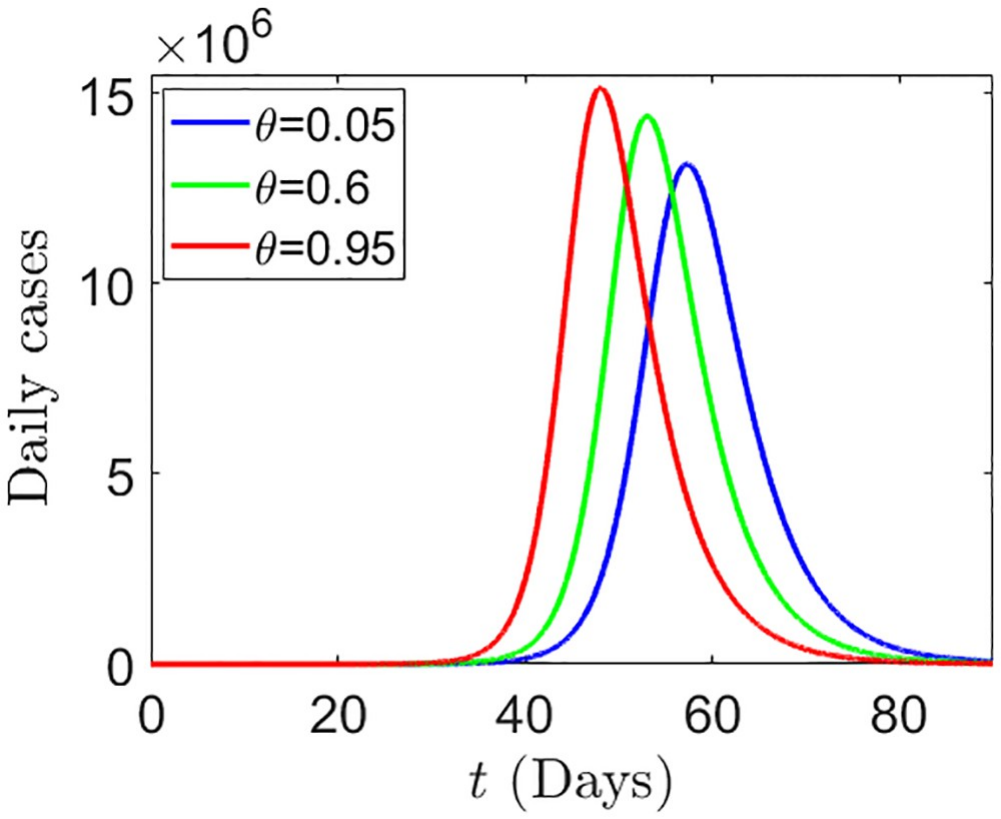
Daily cases for *θ* = 0.05 (blue), *θ* = 0.6 (green), *θ* = 0.95 (red). We assume that *p_u_*(*θ*) = *ρ*(1 − *θ*). The parameter values are: *α_u_* = 1.5, *p_r_* = 0.4, *ρ* = 0.9, *c* = 0.8, *u*(0) = 0.8, and all other parameters are same as before.

**Fig 19:**
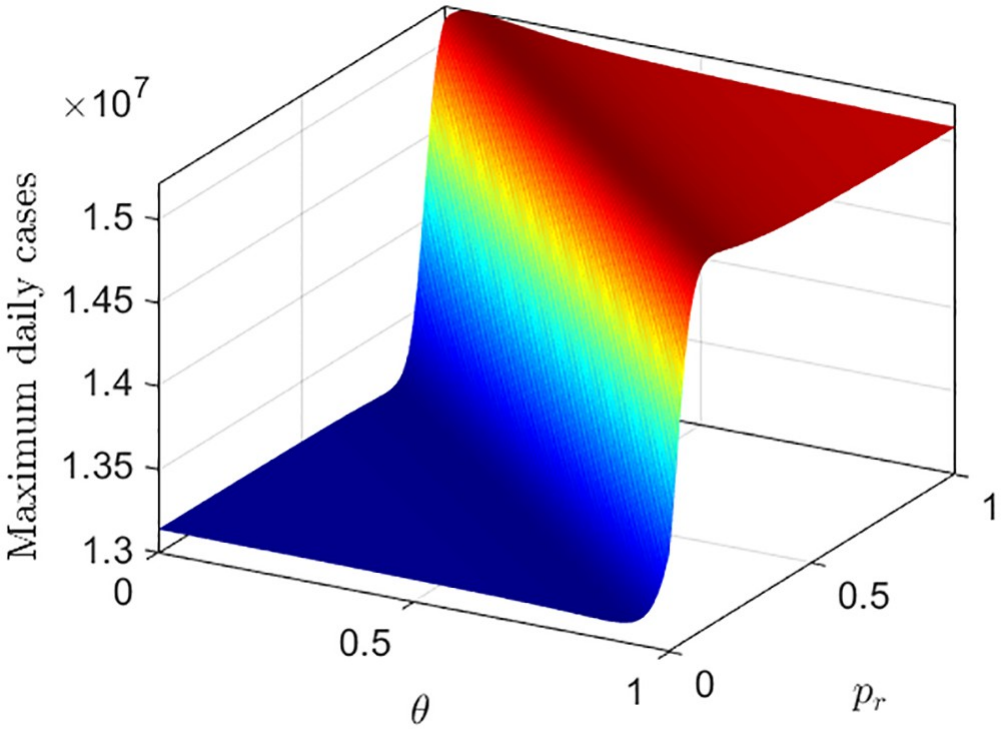
Maximum of daily cases is plotted as a function of *p_r_* and *θ*. We assume that *p_u_*(*θ*) = *ρ*(1 − *θ*). The parameter values are: *α_u_* = 1.5, *ρ* = 0.9, *c* = 0.8, *u*(0) = 0.8, and all other parameters are same as before.

For these three possible scenarios, the simulated results for the daily cases are shown in Fig 18, where, Scenario-I, Scenario-II, and Scenario-III correspond to the blue, green and red curves respectively, in Fig 18. Although, the death data has no direct influence on disease progression, still, Fig 18 shows that, the suppression of death data can influence the epidemic progression implicitly through human behavior. In Fig 18, the red curve which correspond to the suppression of 95% of the death data, gives a higher peak, whereas, the blue curve which correspond to the suppression of only 5% of the death data can reduce the height of the epidemic peak. In Fig 19, we plot the maximum of daily cases as a function of *p*_*r*_ and *θ*, which explains the effect of both *p*_*r*_ and *θ* in determining the maximum height of the epidemic.

## 6 Discussion

In the late 2019, the novel coronavirus SARS-CoV-2 emerged in China and rapidly spread to other parts of the world. To control the transmission of the disease, China implemented strict measures through the zero-COVID policy. With positive intentions, zero-COVID policy aimed to completely eliminate the transmission of COVID-19 in China. Apart from the social and economic issues, another drawback of China’s zero-COVID policy was the low level of immunity achieved due to negligible infection, which prevented the country from attaining acquired immunity in its population [35]. This low immunity left a significant portion susceptible to the virus, contributing to the subsequent surge in cases after policy relaxation. Towards the end of 2022, China began gradually relaxing its zero-COVID policy and the reports in the media suggested a subsequent rapid spread of epidemic [3–6].

Our study aims to investigate the factors contributing to a large outbreak observed in China in the beginning of 2023. An SEIR-type epidemic model is developed that incorporates a time-dependent level of immunity, taking into account the number of vaccine doses administered and the efficacy of the vaccine.

The sudden outbreak following the relaxation of the zero-COVID policy in early November 2022 can be explained by the controlled reproduction number ($R_{c}$) at that time *t* = *t*_1_ (beginning of the outbreak in early November 2022 in China). The expression of $R_{c}$ in (10) shows that $R_{c}$ is inversely proportional to the level of immunity *m*(*t*). Without a zero-COVID policy and a significant prior infection in the population, the level of immunity at time *t*_1_ would be as follows:

$$\tilde{m}\left(t_{1}\right)=m\left(t_{1}\right)+\frac{1}{N}\int_{0}^{t_{1}}\psi \left(t_{1}-\eta \right)R^{\prime }\left(\eta \right)d\eta \;>\;m\left(t_{1}\right),$$

where, *ψ*(*ξ*) is the efficacy of the acquired immunity which depends on the time-post-recovery *ξ* and *R*′(*η*) is the daily new recovery at time *η*. Consequently, the immunity level without the zero-COVID policy $\tilde{m}\left(t_{1}\right)$, would exceed the immunity level with the policy *m*(*t*_1_). This would lead to a comparatively lower value of $R_{c}$ in case of no zero-COVID policy, which indicates a lower peak of the outbreak. However, the use of acquired immunity of recovered to achieve herd immunity in the population is advisable only when the infection is less fatal (e.g., Omicron).

Immunity waning is an important factor which leads to a variation in the level of immunity in the population and consequently it has a significant influence in the emergence of new outbreaks. This immunity waning is considered in our definition of *m*(*t*) (see formula (2)) by the time post vaccination dependent vaccine efficacy. A vaccinated individuals do not immediately reach maximum immunity after vaccination, but rather experience a gradual increase, peak, and subsequent decrease in immunity. This variation is captured through acquisition fading kernels in our model (see formula (14)).

The level of immunity in the population is also influenced by the dynamics of vaccination, for which we directly utilize available data sources. Our approach avoids the introduction of an additional compartment in the model to represent vaccination dynamics, simplifying the model while still considering the impact of vaccination on immunity levels.

Additionally, our model incorporates the imitation dynamics of individuals switching strategies based on net payoffs. Notably, our study explores the implicit effect of under-reporting in death data on individuals’ decision-making processes. To the best of our knowledge, there is no existing mathematical study that explicitly addresses this aspect, highlighting the novelty of our research.

The modeling results of the study demonstrate that time-post-vaccination dependent vaccine efficacy plays a significant role in determining the size of the epidemic outbreak. Also, this work describes the fact that accurate and transparent reporting of daily cases and deaths is crucial to ensure individuals maintain a realistic understanding of the situation. In conclusion, the study emphasizes the importance of considering time-dependent vaccine efficacy and accurate reporting of daily cases and deaths in understanding and controlling the magnitude of an outbreak. The findings highlight the need for highly effective vaccines with durable immunity and transparent reporting systems to effectively combat the spread of COVID-19 and minimize the impact on public health and the economy. In addition, we introduce a novel neural network-based algorithm, specifically a Physics Informed Neural Networks (PINNs), to quantify parameters in the model by learning from the data. This algorithm aids in estimating critical parameters that contribute to understanding the dynamics of the epidemic. We strongly believe that this algorithm will help researchers to estimate many crucial parameters effectively, by learning the data.

The model developed in this work is generic and can be used for other epidemic diseases, but this model is based on several simplifying assumptions due to the lack of specific data. We assumed equal effectiveness for all vaccine doses and did not differentiate between individuals who received the first dose, second dose, or booster dose of the vaccine. Future research can incorporate more accurate data on vaccine efficacy and distinguish between different vaccine doses to calculate immunity levels more precisely. Furthermore, we assumed a homogeneous population with similar immune status for each individual. However, factors such as age can influence an individual’s immune status, and future investigations can extend the model to introduce population heterogeneity through age-structured modeling. Regarding under-reporting, we assumed that the payoff related to under-reporting in infections is a function of the proportion of under-reporting in deaths, represented by *p*_*u*_(*θ*) = *ρ*(1 − *θ*), where *θ* represents the proportion of under-reporting in deaths. Future studies can explore different choices of the function *p*_*u*_(*θ*) to further analyze the impact of under-reporting on individuals’ decision-making.

## Supporting information

S1 Appendix (PDF)
