# Supplementary material for: Understanding the implications of under-reporting, vaccine efficiency and social behavior on the post-pandemic spread using physics informed neural networks: A case study of China
Source: PLoS One. 2023 Nov 16;18(11):e0290368. doi: 10.1371/journal.pone.0290368 (PMC10653536; doi:10.1371/journal.pone.0290368)
Supplement: S1 Appendix — (PDF) [file pone.0290368.s001.pdf]

7 Appendix

7.1 Reproduction Number

We consider the compartments S, R, D as the non-infected compartments and the compartments E, I, A, Q and H as the infected compartments. Then the matrix $\mathcal{F}$ corresponding to the new infection and the matrix $\mathcal{V}$ corresponding to the outflow from the infected compartments are given by:

$$\mathcal{F} = \begin{pmatrix} \frac{\beta S}{N}(I + \alpha A) \\ 0 \\ 0 \\ 0 \\ 0 \end{pmatrix}, \quad \mathcal{V} = \begin{pmatrix} \sigma E \\ (\eta_1 + \eta_2 + \delta_I + \mu_I)I - r\sigma E \\ \delta_A A - (1-r)\sigma E \\ (\xi + \delta_Q)Q - \eta_1 I \\ (\delta_H + \mu_H)H - \eta_2 I - \xi Q \end{pmatrix}.$$

The Jacobian of $\mathcal{F}$ and $\mathcal{V}$ evaluated at the equilibrium point $(S(t_1), 0, 0, 0, 0, 0, 0, 0)$ is given by:

$$F = \begin{pmatrix} 0 & \frac{\beta S(t_1)}{N} & \frac{\alpha\beta S(t_1)}{N} & 0 & 0 \\ 0 & 0 & 0 & 0 & 0 \\ 0 & 0 & 0 & 0 & 0 \\ 0 & 0 & 0 & 0 & 0 \\ 0 & 0 & 0 & 0 & 0 \end{pmatrix},$$

$$V = \begin{pmatrix} \sigma & 0 & 0 & 0 & 0 \\ -r\sigma & (\eta_1 + \eta_2 + \delta_I + \mu_I) & 0 & 0 & 0 \\ -(1-r)\sigma & 0 & \delta_A & 0 & 0 \\ 0 & -\eta_1 & 0 & (\xi + \delta_Q) & 0 \\ 0 & -\eta_2 & 0 & -\xi & (\delta_H + \mu_H) \end{pmatrix}.$$

The reproduction number at time $t = t_1$ is the spectral radius of the matrix FV^{-1} and is given by:

$$\begin{aligned} \mathcal{R}_c &= \frac{\beta S(t_1) r}{N(\eta_1 + \eta_2 + \delta_I + \mu_I)} + \frac{\alpha\beta(1-r)S(t_1)}{\delta_A N} \\ &= (1 - m(t_1))\left(\frac{\beta r}{\eta_1 + \eta_2 + \delta_I + \mu_I} + \frac{\alpha\beta(1-r)}{\delta_A}\right) \\ &= \left(1 - \frac{1}{N}\int_0^{t_1} \phi(t_1 - \eta)V'(\eta)d\eta\right)\left(\frac{\beta r}{\eta_1 + \eta_2 + \delta_I + \mu_I} + \frac{\alpha\beta(1-r)}{\delta_A}\right). \end{aligned}$$

7.2 Under-reporting

The system looks like:

$$\frac{dS}{dt} = -\frac{\beta S}{N}(I_1 + \alpha_u I_2 + \alpha A) - \int_0^t \phi'(t-\eta)V'(\eta)d\eta \quad (16a)$$

$$\frac{dE}{dt} = \frac{\beta S}{N}(I_1 + \alpha_u I_2 + \alpha A) - \sigma E \quad (16b)$$

$$\frac{dI_1}{dt} = ur\sigma E - (\eta_1 + \eta_2 + \delta_I + \mu_I)I_1 \quad (16c)$$

$$\frac{dI_2}{dt} = (1-u)r\sigma E - (\delta_I + \mu_I)I_2 \quad (16d)$$

$$\frac{dA}{dt} = (1-r)\sigma E - \delta_A A \quad (16e)$$

$$
\begin{aligned}
\frac{dQ}{dt} &= \eta_1 I_1 - (\xi + \delta_Q)Q && (16f)\\
\frac{dH}{dt} &= \eta_2 I_1 + \xi Q - (\delta_H + \mu_H)H && (16g)\\
\frac{dR}{dt} &= \delta_I(I_1 + I_2) + \delta_A A + \delta_H H + \delta_Q Q && (16h)\\
\frac{dD}{dt} &= \mu_I(I_1 + I_2) + \mu_H H, && (16i)
\end{aligned}
$$

7.2.1 Controlled reproductive number

We consider the compartments S, R, D as the non-infected compartments and the compartments E, I_1, I_2, A, Q and H as the infected compartments. Then the matrix $\mathcal{F}$ corresponding to the new infection and the matrix $\mathcal{V}$ corresponding to the outflow from the infected compartments are given by:

$$
\mathcal{F} = \begin{pmatrix} \frac{\beta S}{N}(I_1 + \alpha_u I_2 + \alpha A) \\ 0 \\ 0 \\ 0 \\ 0 \\ 0 \end{pmatrix}, \quad \mathcal{V} = \begin{pmatrix} \sigma E \\ (\eta_1 + \eta_2 + \delta_I + \mu_I)I_1 - ur\sigma E \\ (\delta_I + \mu_I)I_2 - (1-u)r\sigma E \\ \delta_A A - (1-r)\sigma E \\ (\xi + \delta_Q)Q - \eta_1 I_1 \\ (\delta_H + \mu_H)H - \eta_2 I_1 - \xi Q \end{pmatrix}.
$$

The Jacobian of $\mathcal{F}$ and $\mathcal{V}$ evaluated at the equilibrium point $(S(t_1), 0, 0, 0, 0, 0, 0, 0)$ is given by:

$$
F = \begin{pmatrix} 0 & \frac{\beta S(t_1)}{N} & \frac{\alpha_u \beta S(t_1)}{N} & \frac{\alpha \beta S(t_1)}{N} & 0 & 0 \\ 0 & 0 & 0 & 0 & 0 & 0 \\ 0 & 0 & 0 & 0 & 0 & 0 \\ 0 & 0 & 0 & 0 & 0 & 0 \\ 0 & 0 & 0 & 0 & 0 & 0 \\ 0 & 0 & 0 & 0 & 0 & 0 \end{pmatrix},
$$

$$
V = \begin{pmatrix} \sigma & 0 & 0 & 0 & 0 & 0 \\ -ur\sigma & (\eta_1 + \eta_2 + \delta_I + \mu_I) & 0 & 0 & 0 & 0 \\ -(1-u)r\sigma & 0 & (\delta_I + \mu_I) & 0 & 0 & 0 \\ -(1-r)\sigma & 0 & 0 & \delta_A & 0 & 0 \\ 0 & -\eta_1 & 0 & 0 & (\xi + \delta_Q) & 0 \\ 0 & -\eta_2 & 0 & 0 & -\xi & (\delta_H + \mu_H) \end{pmatrix}.
$$

The reproduction number at time $t = t_1$ is the spectral radius of the matrix FV^{-1} and is given by:

$$
\mathcal{R}_c^1 = \frac{\beta S(t_1)ur}{N(\eta_1 + \eta_2 + \delta_I + \mu_I)} + \frac{\alpha_u \beta (1-u) r S(t_1)}{\delta_I + \mu_I} + \frac{\alpha \beta (1-r) S(t_1)}{\delta_A N}
$$

$$
\begin{aligned}
&= (1 - m(t_1))\left(\frac{\beta ur}{\eta_1 + \eta_2 + \delta_I + \mu_I} + \frac{\alpha_u \beta (1-u) r}{\delta_I + \mu_I} + \frac{\alpha\beta(1-r)}{\delta_A}\right) \\
&= \left(1 - \frac{1}{N}\int_0^{t_1} \phi(t_1 - \eta) V'(\eta) d\eta\right)\left(\frac{\beta ur}{\eta_1 + \eta_2 + \delta_I + \mu_I} + \frac{\alpha_u \beta (1-u) r}{\delta_I + \mu_I} + \frac{\alpha\beta(1-r)}{\delta_A}\right).
\end{aligned}
$$
